# Nek1 defines a branch of centriolar microtubule length control parallel to CP110-Cep97

**DOI:** 10.1038/s41467-026-73560-9

**Published:** 2026-06-09

**Authors:** J. M. S. Streubel, O. R. Karasu, I. M. Munoz, A. Neuner, E. N. Numanoglu, T. J. Macartney, E. Schiebel, J. Rouse, G. Pereira

**Affiliations:** 1 Centre for Organismal Studies (COS), University of Heidelberg, Heidelberg, Germany; 2 German Cancer Research Centre (DKFZ), DKFZ-ZMBH Alliance, Heidelberg, Germany; 3 MRC Protein Phosphorylation and Ubiquitylation Unit, Faculty of Life Sciences, University of Dundee, Dundee, UK; 4 Division of Genome Integrity, Faculty of Life Sciences, University of Dundee, Dundee, UK; 5 Centre for Molecular Biology (ZMBH), DKFZ-ZMBH Alliance, University of Heidelberg, Heidelberg, Germany

**Keywords:** Centrosome, Microtubules, Ciliogenesis

## Abstract

Centrioles maintain a characteristic length throughout the cell cycle, which is essential for the accurate functioning of centrosomes and ciliogenesis. The CP110-Cep97 complex acts as a cap on the distal end of the centriole, restricting microtubule extension. However, whether CP110-Cep97 alone or in conjunction with additional players regulates this process remains unclear. In this study, we identify the kinase Nek1 (NIMA-related kinase 1) as a key factor that works with the CP110-Cep97 complex to control centriole length. Nek1 localizes alongside CP110 and Cep97 at the distal end of the centriole and interacts with Cep97 and Cep78. Loss of Nek1 induces pronounced centriolar microtubule hyperelongation without displacement of the CP110-Cep97 complex, indicating that Nek1 restricts centriole extension through a distinct mechanism. Co-depletion of Nek1 and CP110, but not Cep78, further enhances the hyperelongation phenotype, demonstrating that Nek1 and CP110 pathways act in parallel to maintain centriole length in cycling cells. Notably, Nek1 is removed from the basal body during ciliogenesis in a Cep78-dependent manner, thereby linking Cep78 to the spatial regulation of Nek1 activity. Together, these findings establish Nek1 as an important safeguard that works with the CP110-Cep97 complex to ensure the structural integrity of centrioles.

## Abstract (web summary)

Proper control of centriole length is essential for centrosome function and ciliogenesis. This study identifies Nek1 as a regulator that cooperates with the CP110-Cep97 complex to maintain centriolar microtubule length in proliferating cells.

## Introduction

Centrosomes are the main microtubule (MT)-organizing centers of mammalian cells. They are directly involved in forming the mitotic spindle and in building cilia, which are MT-based organelles essential for embryonic development and tissue homeostasis^1,2^. Each centrosome contains two centrioles, barrel-shaped structures composed of nine MT triplets, connected at their proximal ends by a proteinaceous linker and surrounded by pericentriolar material. The two centrioles are largely similar but show some differences in composition and function, as only the mother centriole carries large proteinaceous complexes, known as subdistal (SDA) and distal (DA) appendages, which are involved in cell cycle progression and ciliogenesis^2,3^. The primary cilium is a sensory organelle that typically occurs once per cell. Ciliogenesis is tightly coupled to cell cycle progression. In G_0_/G_1_, the mother centriole converts into a basal body via remodeling of protein complexes and assembly of the ciliary membrane, which enables axoneme extension from the centriolar MTs. Later, the cilium is resorbed as the cell re-enters the proliferative cycle^4^.

Centrosome numbers must be tightly controlled to prevent mitotic spindle abnormalities and chromosome mis-segregation as a result of centrosome overamplification. During the G_1_/S phase, the centrosome duplicates, ensuring that each daughter cell inherits a complete centrosome after mitosis. The kinase Plk4 initiates this process by recruiting Sas6, the core protein of the centriole cartwheel. Sas6, in turn, recruits Cep135 and CPAP, an essential protein for procentriole formation and elongation^5^. Excessive CPAP expression causes centriolar MTs to hyperelongate, resulting in cell cycle defects^6,7^.

The length of centrioles is a critical determinant of their function and varies across cell types and growth conditions. Although several mechanisms that control centriole length have been identified^8^, how these mechanisms operate and are regulated, and whether additional mechanisms exist, remains poorly understood. It is known that CPAP binds to the plus ends of centriole MTs to regulate their length. CPAP inhibits catastrophes and promotes rescue events, thereby stabilizing MTs. At the same time, its capping of centriolar MTs restricts further polymerization, resulting in slow MT elongation^9,10^. In addition, two protein complexes, the DISCO (distal centriolar complex) and the distal tip complex, reside at the centriolar distal end and contribute to centriole length control. DISCO complex components (Cep90, MNR, and OFD1) localize to both the centriolar distal tip (slightly below the distal end, at the same level as DAs^11,12^), and centriolar satellites^12^. Centriolar satellites are large cytoplasmic proteinaceous granules that deliver client proteins to centrosomes for centriole duplication and cilia formation^13^. While all components of the DISCO complex at the centriole tip are required for DA assembly, OFD1 also restricts centriole elongation^14^.

The distal tip complex is an evolutionarily conserved protein complex that contains Kif24, the CP110-Cep97 complex, and Cep104. Cep104 is recruited to nascent centrioles by Cep97 and considered as a key regulator of centriole length^15^. The loss of either CP110 or Cep97 has been shown to enhance ciliogenesis, and their removal is required for axoneme extension^16^. Lack of CP110 was also shown to cause centriolar MTs to hyperelongate^7,17,18^. However, this phenotype seems to be organism- and tissue-specific, since other studies were unable to reproduce it^19^. Kif24 does not by itself influence centriole length but acts by stabilizing CP110 mainly at the mother centriole, thereby affecting ciliogenesis^20^. CP110 levels are controlled by the centriolar tip protein Cep78 through binding of Cep78 to the E3 ubiquitin ligase EDD-DYRK2-DDB1^VprBP^^21,22^. Interestingly, while Goncalves *et al*. (2021) reported that Cep78 recruits the E3 ubiquitin ligase to the centrosome, promoting CP110 degradation^22^, Hossain *et al*. (2017) found that the interaction of this complex with Cep78 inhibits CP110 ubiquitination and degradation^21^. Thus, the exact role of Cep78 in CP110 regulation remains unclear.

The Nek (NIMA-related kinase) family is a highly conserved group of serine/threonine kinases. In humans, there are eleven Nek kinases (Nek1-11)^23^. Despite their established role in tumor progression^24^, Nek kinases remain understudied. Their functions range from cell cycle regulation and DNA damage response (DDR) to centrosome separation, ciliogenesis, and maintenance of mitochondria^23,25^. Nek1 has been implicated in homology-directed DDR, cytokinesis, and MT stability^26–32^. *NEK1* overexpression in mouse embryonic fibroblasts significantly reduces the ability of cells to ciliate^31^. In humans, a mutation in the *NEK1* gene has been associated with the ciliopathy autosomal-recessive short-rib polydactyly syndrome Majewski type^33,34^. Deletion of Nek1 in human cells impairs ciliogenesis^35^, but the mechanisms remain incompletely understood.

Building on previous reports that Nek1 functions at the centrosome^30,31^, we aimed to analyze its centrosomal function in human retinal epithelial cells. We show that Nek1 localizes at the distal tip of centrioles throughout the cell cycle, and its loss results in hyperelongation of centriolar MTs from the distal tip. Our data is consistent with a model in which Nek1 and CP110 pathways act synergistically to maintain centriole length in cycling cells, establishing Nek1 as a safeguard of centriole architecture.

## Results

### Nek1 localizes at the distal tip of mother and daughter centrioles in cycling cells

**Fig. 1:**
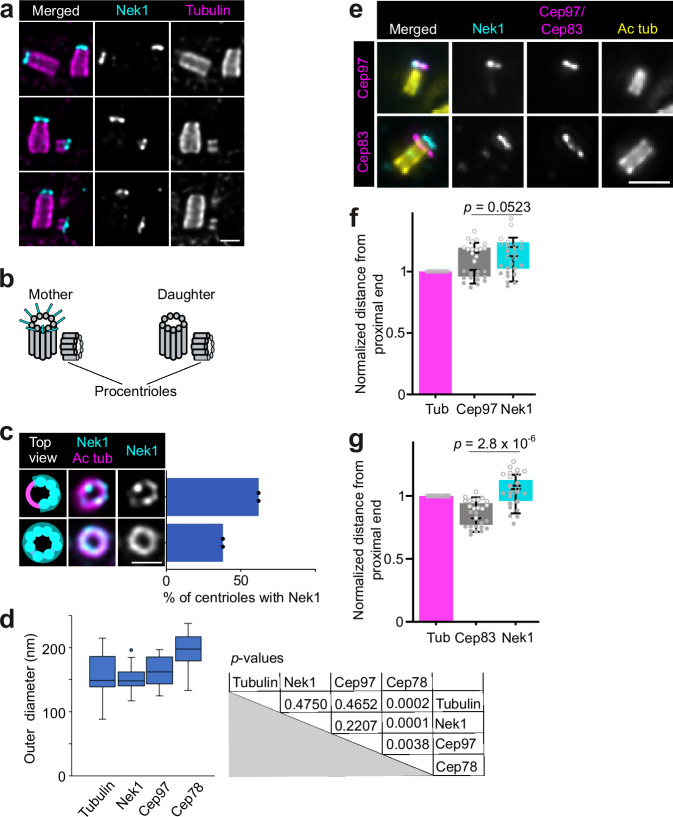
Nek1 localizes to the centriolar distal tip. **a-b** Analysis of Nek1 localization at mother, daughter, and procentrioles, as depicted in the cartoons shown in (**b**). Representative U-ExM images of centrosomes in RPE1 cells in different phases of procentriole formation from three independent biological replicates (**a**). Centrioles were stained for Nek1 and α-tubulin (centriolar marker). Scale bar, 250 nm. **c** Top-view representative U-ExM images of Nek1 at centrioles (acetylated tubulin, Ac tub) in cycling RPE1 cells. The graphs indicate the percentage of centrioles displaying incomplete or complete circular Nek1 staining according to the left panels. The quantification shows the average +/– SD of 42 centrioles from two independent biological replicates. Scale bar, 250 nm. **d** Measurement of the outer diameter of tubulin and distal centriolar proteins by U-ExM. The median, upper and lower quartiles of two independent biological replicates per distal tip protein are shown. Tubulin *n* = 43, Nek1 *n* = 14, Cep97 *n* = 13, Cep78 *n* = 16. Maximum, upper whisker, median, lower whisker, and minimum values (in nm) are as follows: Tubulin: 214.15, 214.15, 148.66, 88.40, 88.40; Nek1: 195.91, 184.56, 147.77, 116.85, 116.85; Cep97: 196.75, 196.75, 161.31, 124.48, 124.48; Cep78: 238.03, 238.03, 197.08, 133.06, 133.06. The table shows p-values calculated using a two-tailed, unpaired Student’s *t*-test. **e** U-ExM showing the relative localization of Nek1 with Cep97 or Cep83 at the centriole (acetylated tubulin, Ac tub) in RPE1 cells. Scale bar, 500 nm. **f**, **g** Quantification of (**e**). The distance from the centriolar proximal end normalized to the centriole length (tubulin, Tub) is shown. The lower and the upper signal boundaries are indicated by filled and open circles, respectively. Average +/− SD of two (**f**) or three (**g**) independent biological replicates. *n* = 14 cells in both graphs. Statistics (two-tailed, unpaired Student’s t test) are based on the lower boundary values. Source data are provided as a Source Data file.

Nek1 has been reported to localize to the centrioles of both ciliated and non-ciliated cells throughout the cell cycle^30,31^. However, its specific centriolar sub-localization remains unknown. To determine Nek1’s precise localization at the centrosome, we performed ultrastructure expansion microscopy (U-ExM) in human retinal pigment epithelial cells (RPE1) using a previously characterized Nek1 antibody^35^. In the G_1_ phase of the cell cycle, Nek1 was detected at the distal tip of both mother and daughter centrioles (Fig. 1a, b). Centriolar tip localization of Nek1 was also observed in ARPE-19, a spontaneously arising immortalized human retinal pigment epithelial cell line^36^ (Supplementary Fig. 1a, b). Importantly, the Nek1 signal was specific, as it disappeared in ARPE-19 cells lacking *NEK1*^35^ (Supplementary Fig. 1a), and a similar localization pattern was observed using a commercially available anti-Nek1 antibody (Supplementary Fig. 1b).

Top-view images revealed that Nek1 formed dot-like structures at the centriolar tip, arranged in a C-shape (Fig. 1c, upper panel) or a complete ring (Fig. 1c, lower panel). A similar pattern was observed with a commercially available anti-Nek1 antibody (Supplementary Fig. 1c). Comparison of the Nek1 ring diameter with that of tubulin and two additional distal components (Cep78 and the centriolar capping protein Cep97) showed that Nek1 formed rings of similar size to centriolar MTs and Cep97, and smaller than those formed by Cep78 (Fig. 1d), indicating that Nek1 localizes close to the centriolar MT wall. In side views, Nek1 localized similarly to Cep97 at the centriolar distal tip, but more distally than the DA component Cep83 (Fig. 1e-g). This prompted us to postulate that Nek1 associates with centrioles independently of DAs or SDAs. Indeed, the percentage of Nek1-positive centrioles in cells that are unable to assemble specifically DAs (*CEP83* KO), SDAs (*ODF2* KO^37^), or both (*CEP350* KO^38^), did not differ in comparison to the control (Supplementary Fig. 1d), indicating that Nek1 does not associate with centrioles via appendages.

To understand the timing of Nek1 recruitment to centrioles, we inspected Nek1 localization during procentriole formation, which occurs during the S phase of the cell cycle^5^. Nek1 associated with procentrioles from the early stages of procentriole assembly, concomitant with the recruitment of tubulin (Fig. 1a, lower panel), and remained at the centriole tip in longer procentrioles (Fig. 1a). Nek1 localized at both centrioles in all stages of the cell cycle (Supplementary Fig. 1e).

Together, our data indicate that Nek1 localizes to the distal tip of both mother and daughter centrioles throughout the cell cycle.

### Absence of Nek1 results in hyperelongated centriolar microtubules

**Fig. 2:**
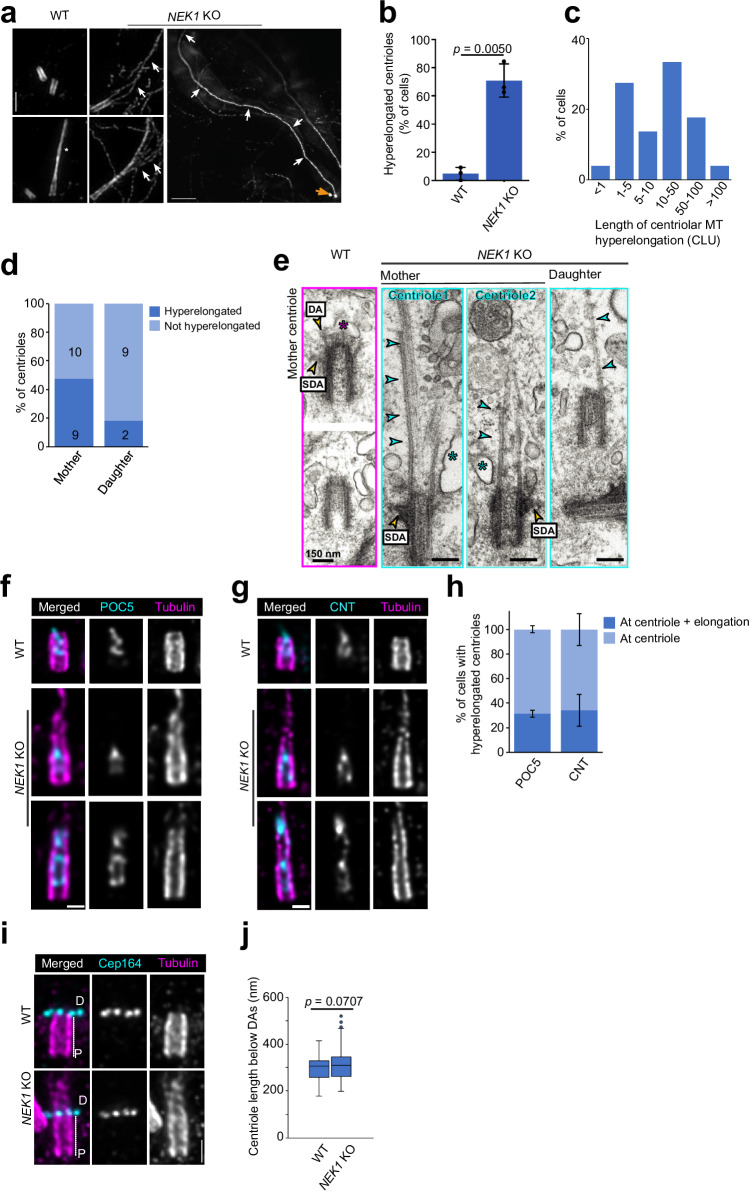
Centrioles in ARPE-19 *NEK1* KO cells hyperelongate from the distal tip. **a** U-ExM images of centrioles in ARPE-19 cells. Asterisk in WT cells: cilium, white arrows: elongations of centrioles in *NEK1* KO cells, orange arrow: centrosome. Scale bar, 500 nm. Scale bar (overview image), 1 μm. **b** Quantification of the cells displaying at least one hyperelongated centriole from (**a**). Average +/– SD of three independent biological replicates. WT n = 92, *NEK1* KO n = 105. Statistics show two-tailed, unpaired Student’s t-test. **c** Estimation of the length of centriolar hyperelongations compared to the length of the centriole core from the proximal end to the DAs (centriole length unit, CLU). Quantifications from hyperelongated centrioles of four independent biological replicates. *n* = 51. **d** Percentage of cells with hyperelongated mother and daughter centrioles in ARPE-19 *NEK1* KO cells quantified by transmission electron microscopy. Numbers in the bars: number of cells showing the respective phenotype. Mother *n* = 19, daughter *n* = 11. **e** Transmission electron micrographs showing centrioles in cycling cells from (**d**). Magenta, WT, cyan: *NEK1* KO. Magenta/cyan arrowheads: hyperelongated MTs, yellow arrowheads: distal appendages (DA) or subdistal appendages (SDA) as indicated, asterisks: vesicles. Scale bar, 150 nm. **f-g** U-ExM images of POC5 (**f**) and Centrin (CNT) (**g**) at the centrioles (α-tubulin) of ARPE-19 WT and *NEK1* KO cells. Scale bar, 200 nm. **h** Quantification of (**f**) and (**g)** showing the number of hyperelongated centrioles in *NEK1* KO cells with POC5 and Centrin (CNT) present at the centriole core only or at the core plus hyperelongations. Average +/− SD of two independent biological replicates. POC5 *n* = 23, Centrin *n* = 31. **i-j** Side view images (**i**) and size quantification (**j**) of expanded mother centrioles of ARPE-19 WT and *NEK1* KO cells (Cep164 and α-tubulin). The distance from the proximal end (P) to the distal appendages (DA) (as depicted in (**i**)) was determined. Scale bar, 250 nm. **j** shows the median, upper and lower quartiles of two independent biological replicates. Maximum, upper whisker, median, lower whisker, and minimum values (in nm): WT: 413.43, 413.43, 305.45, 178.10, 178.10; *NEK1* KO: 520.32, 474.19, 310.74, 198.66, 198.66. Statistics show a two-tailed, unpaired Student’s *t*-test. WT *n* = 41, *NEK1* KO *n* = 52. Source data are provided as a Source Data file.

To characterize the role of Nek1 at the distal tips of centrioles, we made use of an established *NEK1* knockout (KO) cell line generated in ARPE-19^35^. Centriolar MTs in ARPE-19 WT and *NEK1* KO cells were stained using acetylated tubulin and analyzed by U-ExM. Surprisingly, the results revealed that over 60% of *NEK1* KO cells exhibited hyperelongated centriolar MTs (Fig. 2a, b). The hyperextension phenotype upon *NEK1* loss was complemented by stably expressing a WT *NEK1* construct in *NEK1* KO cells^35^ (Supplementary Fig. 2a), confirming that the observed hyperextensions are Nek1 dependent. Interestingly, a significant increase in the percentage of cells displaying centriolar MT hyperelongation was also observed in *NEK1* KO cells complemented with the *NEK1* kinase-dead mutant (Nek1-KD; D146A^35^) in comparison to WT or *NEK1* KO + *NEK1* cells (Supplementary Fig. 2b, c), indicating that Nek1 kinase activity is involved in preventing centriolar MT hyperelongation. However, as the *NEK1* + *NEK1-KD* phenotype was milder than the *NEK1* KO phenotype (Supplementary Fig. 2c), we cannot exclude that the kinase-independent function of Nek1 is also involved.

Extension of centriolar MTs occurs during axoneme formation in ciliogenesis. In a small percentage of ARPE-19 WT cells growing under serum-rich conditions (10–20%), axonemal MTs appeared as focused extensions of centriolar MTs, forming a single, continuous MT-based structure (Fig. 2a, asterisk). In contrast, a significantly higher proportion (>60%) of ARPE-19 *NEK1* KO cells displayed randomly extended, disconnected MT filaments emanating from the centrioles (Fig. 2a, white arrows). MT extensions displayed substantial length variability across cells, making precise absolute measurements difficult. To assess their length distribution, extension length was normalized to the centriolar core, defined as one centriole length unit (CLU). While some extensions measured less than 5 CLUs, a considerable fraction reached 10–50 or even 100 CLUs (Fig. 2c).

Unlike cilia, which are mainly present at the G_1_/G_0_ phase of the cell cycle^4^, centriolar hyperextensions in *NEK1* KO cells were also present even in M phase cells (Supplementary Fig. 2d). Importantly, in contrast to WT cells, the large majority of centrioles in cycling *NEK1* KO cells (>99%) was not associated with a cilium, based on staining with the ciliary membrane marker Arl13b (Supplementary Fig. 2e, f). To further exclude the possibility that the *NEK1* KO phenotype arises from unscheduled or incomplete ciliogenesis, we depleted Cep123, a DA protein essential for the initial steps of cilia formation^39^. Depletion of Cep123 by small interference RNA (siRNA) did not affect the percentage of *NEK1* KO cells displaying hyperextended MTs (Supplementary Fig. 2g–i), indicating that centriolar MT hyperelongation occurs independently of ciliogenesis.

Next, we employed transmission electron microscopy (TEM) to obtain high-resolution images of centrioles from cycling WT and *NEK1* KO cells. Longitudinal sections of the centrioles showed that *NEK1* KO cells exhibited abnormally extended MTs, which appeared to emanate from the distal tips of the centrioles (Fig. 2d, e). Vesicles (Fig. 2e, asterisks) were observed in the vicinity of both WT and *NEK1* KO centrioles. In serum-starved cells, ciliary vesicles ensheathed the tips of mother centrioles in WT cells, but not in *NEK1* KO cells (Supplementary Fig. 2j), confirming the absence of a ciliary membrane along extended centriolar MTs in the absence of Nek1, even under cilia-inducing conditions.

To clarify whether Nek1 absence affected centriolar body length, we stained the centrioles for Centrin and POC5, which are proteins known to extend together with centrioles if the main centriole core length increases^38^. Our results showed that the localization of Centrin and POC5 did not differ between WT and *NEK1* KO in the majority of cells, yet 25% of *NEK1* KO centrioles had Centrin and POC5 elongating together with hyperextending MTs (Fig. 2f–h). When we measured the distance between the proximal end of centrioles to the DA marker Cep164^40^, we did not observe any significant increase in centriolar length compared to control cells (Fig. 2i, j), indicating that loss of Nek1 does not result in elongated core centrioles.

Overall, our results show that Nek1 prevents centriole MT extensions from the tips of the centrioles rather than controlling the main centriole core length.

### Nek1 functions primarily at the mother centrioles

**Fig. 3:**
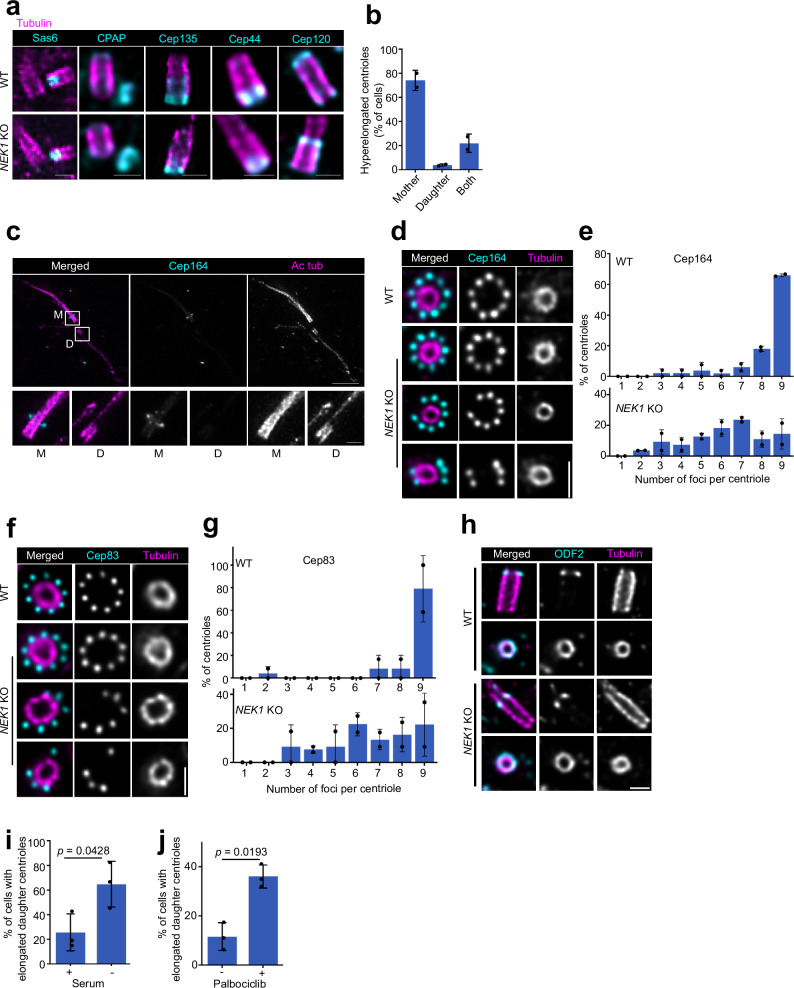
Mother centrioles hyperelongate faster than daughters. **a** Representative U-ExM images from two independent biological replicates for proteins localized at the proximal end of the centrioles in ARPE-19 WT and *NEK1* KO cells. See Supplementary Fig. 3 for single channels. Scale bar, 250 nm. **b** Quantification of the percentage of ARPE-19 *NEK1* KO cells showing hyperelongated daughter centrioles by U-ExM. Average +/− SD of two independent biological replicates. *n* = 52 cells. **c** Representative U-ExM showing centrioles in ARPE-19 *NEK1* KO cells with mother (M) and daughter (D) centrioles hyperelongated. Centrioles: acetylated tubulin (Ac tub), mother centriole: Cep164. Scale bar: overview 1250 nm; enlargements, 250 nm. **d** Top-view U-ExM images of Cep164 at mother centrioles (α-tubulin) in ARPE-19 WT or *NEK1* KO cells. Scale bar, 250 nm. **e** Quantification of (**d**) showing the number of Cep164 densities around mother centrioles from top-view images. Average +/− SD of two independent biological replicates. WT *n* = 50, *NEK1* KO *n* = 55. **f** Top-view U-ExM images of Cep83 at mother centrioles (α-tubulin) in ARPE-19 WT or *NEK1* KO cells. Scale bar, 250 nm. **g** Quantification of (**f**) showing the number of Cep83 densities around mother centrioles from top-view images. Average +/− SD of two independent biological replicates. WT *n* = 27, *NEK1* KO *n* = 28. **h** Side and top-view U-ExM images of ODF2 at the centrioles (α-tubulin) in ARPE-19 WT and *NEK1* KO cells. Scale bar, 250 nm. **i** ARPE-19 *NEK1* KO cells were serum-deprived for 48 h prior to U-ExM. Hyperelongated centrioles were quantified by acetylated tubulin. Only cells with at least one hyperelongated centriole were considered for quantification. Average +/− SD of three independent biological replicates. Plus (+) serum *n* = 69, minus (−) serum *n* = 75. Statistics are based on a paired, one-tailed Student’s *t*-test. **j** ARPE-19 *NEK1* KO cells were treated with 1 μM Palbociclib (+) or DMSO solvent control (−) for 48 h prior to U-ExM. Hyperelongated centrioles were quantified by acetylated tubulin. Only cells with at least one hyperelongated centriole were considered for quantification. Average +/− SD of three independent biological replicates. Palbociclib (+) *n* = 62, Control (−) *n* = 48. Statistics are based on a paired, one-tailed Student’s *t*-test. Source data are provided as a Source Data file.

Given that Nek1 localizes to early procentriole seeds, we next asked whether it plays a role in centriole duplication in S phase^5^. For this, we stained newly synthesized DNA with the thymidine analog EdU to label cells in S phase^41^. WT and *NEK1* KO cells formed procentrioles to a similar extent during S phase (Supplementary Fig. 3a, b), arguing against a failure in centriole duplication. By U-ExM in S phase cells, 100% of WT (*n* = 32) and 96% of *NEK1* KO (*n* = 26) cells formed no more than one procentriole per pre-existing centriole, indicating no centriole overduplication. To further investigate procentriole formation, we employed U-ExM to analyze markers involved in early stages of procentriole assembly (Fig. 3a, Supplementary Fig. 3c–n). The activation of the kinase Plk4 in early S phase promotes the recruitment of the protein Sas6 for the formation of a cartwheel^42^. Following Sas6, additional centriole components join the growing procentrioles, including the proteins CPAP, Cep120, Cep135 and Cep44, which are all involved in procentriole assembly^43,44^. Except for Sas6, which is degraded from the mother centriole in mitosis^45^, and Cep120, which asymmetrically localizes to the daughter centrioles^46^, CPAP, Cep135, and Cep44 remained associated with the proximal end of mature centrioles (Fig. 3a)^47^. For these procentriole-associated proteins – Sas6 (Supplementary Fig. 3c, d), CPAP (Supplementary Fig. 3e, f), Cep135 (Supplementary Fig. 3g, h), Cep120 (Supplementary Fig. 3i, j), and Cep44 (Supplementary Fig. 3k, l) – we did not observe any differences in centriolar localization patterns compared to control cells, indicating that Nek1 does not play a role in centriole duplication.

Interestingly, U-ExM analysis of *NEK1* KO cells using the DA protein Cep164 as a marker for mother centrioles revealed that hyperelongated MTs extended preferentially from the mother centriole in the majority (75%) of *NEK1* KO cells (Fig. 3b, c). Hyperelongation of both mother and daughter centrioles was observed in 20% of the cells, whereas extensions of only daughter centrioles were rare (<5%) (Fig. 3b, c). Compared to the extensions from mother centrioles, MT extensions from daughter centrioles were asymmetric and thinner (Fig. 3c). These data suggest that mature mother centrioles are more susceptible to MT hyperelongation than daughter centrioles.

DA complexes form nine-fold blade-like structures at the distal end of the mother centriole^48,49^. Images from mother centrioles stained with Cep164 revealed that the number of DA foci was reduced in *NEK1* KO cells (Fig. 3d, e). To be able to confirm this defect, we checked Cep83, which is the first protein to be recruited to the mother centriole during the hierarchical process of DA formation^50,51^. Like Cep164, Cep83 also showed disrupted organization (Fig. 3f, g). However, SDAs were not disorganized, as indicated by ODF2 staining (Fig. 3h). In addition, Cep350 and C2CD3, two proteins required for appendage assembly^38,52^, showed very similar localization on the centrioles of *NEK1* KO cells in comparison to WT control cells (Supplementary Fig. 3m–p). These observations suggest that loss of Nek1 primarily affects DAs without altering the localization of SDA proteins.

Next, we sought to explore the mechanisms underlying the milder *NEK1* KO phenotype observed in daughter centrioles. Key differences between the two centrioles of a pair lie in their relative age and in the presence of appendages. While younger (daughter) centrioles do not carry appendages, the older (mother) centrioles acquire them during the maturation process^2^. One possibility is that mother centrioles are more prone to extend centriolar MTs due to the presence of appendages. However, this hypothesis was previously excluded, as the absence of Cep123 (Supplementary Fig. 2g–i) did not reduce the occurrence of hyperelongated mother centrioles. We therefore tested the alternative hypothesis that mother centrioles exhibit a stronger phenotype simply because they have had more time to develop it as a result of their cell cycle-dependent maturation. We reasoned that if time is a key factor, holding daughter centrioles in a particular cell cycle phase would allow the phenotype to emerge more prominently over time. To test this notion, we arrested cells in G_1_/G_0_ via serum starvation and, alternatively, in G_1_ using the Cdk4/6 inhibitor Palbociclib^53^. In both conditions, we observed a significantly higher percentage of daughter centrioles with hyperelongated MTs (Fig. 3i, j), indicating that the MT hyperextension phenotype becomes more pronounced with prolonged time.

Collectively, these findings indicate that Nek1 is not required for centriole duplication but instead functions in regulating MT elongation at the distal tips of mainly mother centrioles.

### Nek1 forms common complexes with the distal tip proteins Cep97 and Cep78

The distal tip complex, formed by CP110-Cep97, blocks the elongation of centriolar MTs at their plus end during the cell cycle, and its removal is essential for axoneme extension and ciliogenesis^16^. CP110 removal is regulated by Cep78^21,22^. Additionally, the DISCO components MNR and Cep90 are required for centriolar recruitment of OFD1, which in turn restricts centriole length^12,14^

**Fig. 4:**
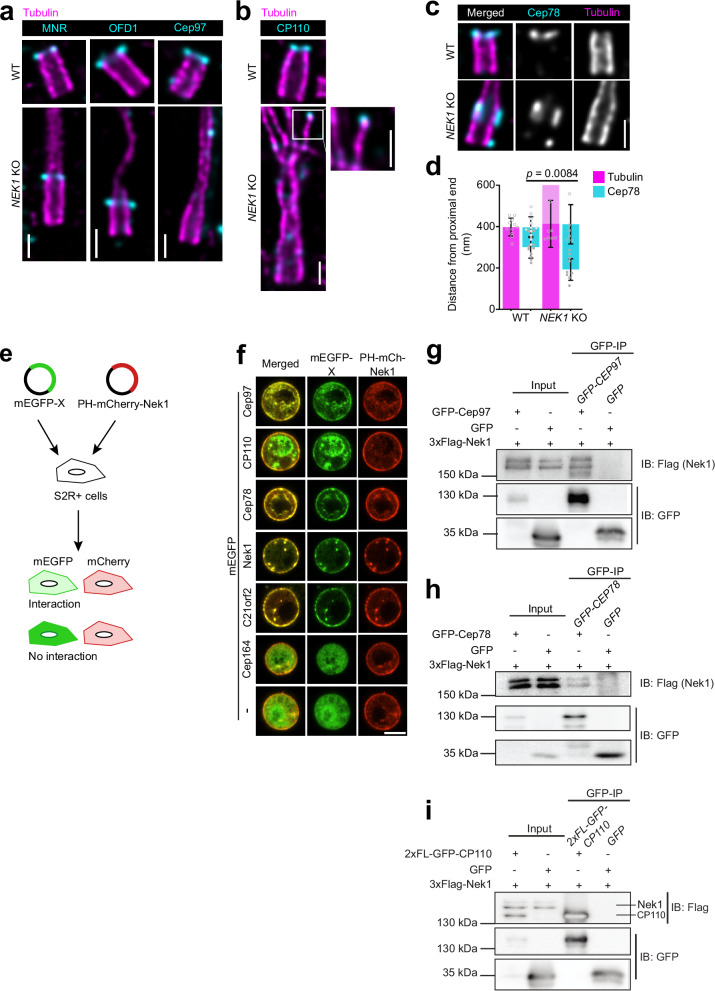
Nek1 interacts with Cep78 and Cep97 at the centriolar distal tip. **a** U-ExM images showing the localization of the centriolar distal tip proteins MNR (two independent biological replicates), OFD1, and Cep97 (three independent biological replicates each) at the centrioles (α-tubulin) of ARPE-19 *NEK1* KO cells. Only merged images are shown; see Supplementary Fig. 4a–c for single channels. Scale bar, 250 nm. **b** U-ExM images of CP110 at the centrioles (α-tubulin) of ARPE-19 WT and *NEK1* KO cells. Representative images from two independent biological replicates are shown. Enlargement is shown as indicated. Only merged images are shown; see Supplementary Fig. 4d for single channels. Scale bar, 250 nm. **c** U-ExM images of Cep78 at centrioles (α-tubulin) in ARPE-19 WT and *NEK1* KO cells. Scale bar, 250 nm. **d** Quantification of Cep78 signal length at the mother centriole in the cells from (**c**). The lower and the upper signal boundaries are indicated by filled and open circles, respectively. Average +/− SD of 11 (WT) or 8 (*NEK1* KO) cells is shown. Statistics are based on a two-tailed, unpaired Student’s *t*-test. **e** Scheme representing the ReLo system. See text for details. **f** ReLo assay for analysis of binary interactions. Representative images of S2R+ cells co-expressing the indicated mEGFP- and PH-mCherry (mCh) constructs from two independent biological replicates are shown. Scale bar, 5 μm. **g-i** Immunoblots (IB) showing co-immunoprecipitations (Co-IP) of 3xFlag-Nek1 with mEGFP-Cep97 (**g**), 2xFlag-mEGFP-CP110 (**h**), and mEGFP-Cep78 (**i**) in Hek293T cells transiently overexpressing both *3xFLAG-NEK1* and EGFP-fusion proteins. Co-IPs were performed in biological triplicates. Source data are provided as a Source Data file.

Based on this, we investigated whether the loss of Nek1 affects the composition or function of centriole tip complexes. To this end, we analyzed the localization of MNR, OFD1, CP110-Cep97, and Cep78 using U-ExM. Our results show that, unlike MNR and OFD1 (Fig. 4a, Supplementary Fig. 4a, b), the CP110-Cep97 complex did not remain at the original centriole tip but instead followed the extending MT plus ends (Fig. 4a, b, Supplementary Fig. 4c–e). Interestingly, Cep78 exhibited a more dispersed centriolar localization in *NEK1* KO cells, although it did not track the extending MT ends but rather elongated towards the proximal end (Fig. 4c, d). These findings suggest that Nek1 restricts Cep78 localization to the centriolar distal tip, and indicate that Nek1 may interact with components of the centriole tip complex to regulate their localization.

To test this idea, we employed a protein-protein interaction assay known as ReLo (ReLocalization)^54^. This system relies on overexpressing a target protein fused to mCherry and a Pleckstrin homology (PH) domain, which anchors the fusion protein to the plasma membrane of *Drosophila melanogaster* S2R+ cells (Fig. 4e). Candidate interaction partners are tagged with mEGFP and co-transfected into the same cells. If an interaction occurs, the mEGFP-tagged candidate is recruited to the plasma membrane together the mCherry-PH-tagged target bait, whereas the absence of membrane re-localization indicates no detectable interaction (Fig. 4e). Using the ReLo system, mCherry-PH-Nek1 was successfully localized to the cell membrane (Fig. 4f, last column). As potential interactors, we tagged *CEP97*, *CP110*, and *CEP78* with *mEGFP*. *mEGFP-C21ORF2*, a known Nek1 interactor^55^, served as a positive control, while *mEGFP-CEP164* and *mEGFP* alone served as negative controls (Fig. 4f). The results revealed positive interactions between Nek1 and CP110, Cep97, and Cep78 (Fig. 4f). mEGFP-Cep97 and mEGFP-Cep78 bound specifically to Nek1, as neither of them bound to the PH-mCherry control (Supplementary Fig. 4f). In contrast, the CP110-Nek1 interaction was inconclusive because mEGFP-CP110 relocated to the plasma membrane also in the absence of Nek1 by binding to PH-mCherry (Supplementary Fig. 4f), suggesting a false-positive signal.

To verify these interactions in human cells, *mEGFP*-tagged *CEP97*, *CP110*, and *CEP78* were separately co-overexpressed with FLAG-tagged *NEK1*. GFP-Trap beads were used to pull down the mEGFP-tagged proteins. Our results showed that Cep97 and Cep78, but not CP110, were able to pull down Nek1 (Fig. 4g–i). These data confirm that Nek1 interacts with Cep97 and Cep78, suggesting that they might be part of common complexes.

### Acute Nek1 depletion promotes centriolar microtubule extension without CP110-Cep97 complex removal

Analysis of CP110 in *NEK1* KO cells revealed that the CP110-Cep97 complex localizes to the extended centriolar MTs (Fig. 4a, b, Supplementary Fig. 4a–e). However, it remains unclear whether the complex is initially removed at the onset of MT extension and subsequently rebinds to the plus ends after elongation, or whether it remains associated with the MT plus ends throughout elongation. This is a crucial point, as CP110-Cep97 removal from the centriole distal end is thought to be a prerequisite for centriole MT elongation^16^.

**Fig. 5:**
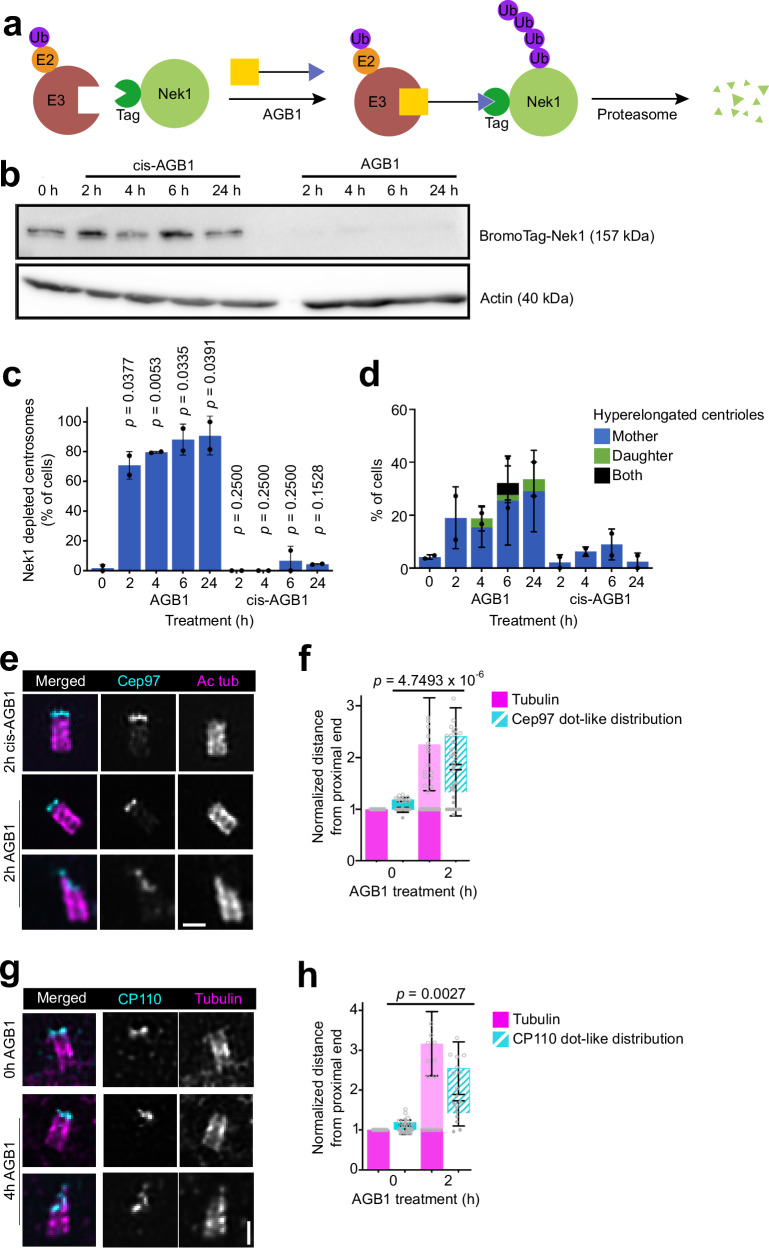
Inducible Nek1 degradation causes rapid hyperelongation of centriolar MTs. **a** Scheme depicting the BromoTag degron strategy. Ub, ubiquitin; E2/E3, ubiquitin ligases. **b** RPE1 *BromoTag-NEK1* cells were treated with AGB1 (Nek1 degradation) or cis-AGB1 (negative control) for the indicated time points. The immunoblot shows BromoTag-Nek1 detected with anti-Nek1 antibodies. Loading control: actin. *N* = 2 independent biological replicates. **c** Presence or absence of Nek1 at the centrosome by U-ExM. Average +/− SD of two independent biological replicates. Number of cells analyzed: 0 h, 50; AGB1 (2, 4, 6 and 24 h): 50, 54, 53, 49; cisAGB1 (2, 4, 6 and 24 h): 51, 47, 43, 45. Statistics are based on a paired, one-tailed Student’s *t*-test. **d** Quantification of (**c**) showing the percentage of RPE1 *BromoTag-NEK1* cells with hyperelongated mother (Cep164) or daughter centrioles (acetylated tubulin, Ac tub). Average +/− SD of two independent biological replicates. Number of cells analyzed: 0 h, 49; AGB1 (2, 4, 6 and 24 h): 50, 54, 46, 47; cisAGB1 (2, 4, 6 and 24 h): 52, 47, 44, 45. Statistics are based on a paired, one-tailed Student’s t-test. Single data points and statistical tests refer to the total percentage of cells displaying at least one hyperelongated centriole. **e** U-ExM showing the localization of Cep97 at the centrioles (acetylated tubulin, Ac tub) in RPE1 *BromoTag-NEK1* cells following 2 h of Nek1 degradation by AGB1. Scale bar, 250 nm. **f** Quantification of the length of Cep97 distribution along the centriole from (**e**). Only hyperelongated centrioles were quantified. Hyperelongations are indicated in pale magenta. Average +/− SD of two (cis-AGB1) or three (AGB1) independent biological replicates. 2 h cis-AGB1 *n* = 14, 2 h AGB1 *n* = 17. Statistics are based on a two-tailed, unpaired Student’s *t*-test. **g** U-ExM showing the localization of CP110 at the centrioles (α-tubulin) in RPE1 *BromoTag-NEK1* cells following 6 h of Nek1 degradation by AGB1. Scale bar, 250 nm. **h** Quantification of the length of CP110 distribution along the centriole in cells from (**g**). Only hyperelongated centrioles were quantified. Hyperelongations are indicated in pale magenta. Average +/− SD of three independent biological replicates. 0 h AGB1 *n* = 14, 2 h AGB1 *n* = 9. Statistics are based on a two-tailed, unpaired Student’s *t*-test. Source data are provided as a Source Data file.

To investigate this, we created a homozygous knock-in RPE1 cell line expressing endogenous *NEK1* fused to the BromoTag (*BromoTag-NEK1*), enabling rapid Nek1 depletion with the BromoTag system^56^. Degradation was induced by the PROTAC (proteolysis targeting chimera) molecule AGB1, while cis-AGB1, which cannot recruit the E3 ubiquitin ligase von Hippel-Lindau (VHL), served as a negative control^56^ (Fig. 5a). Our results show that Nek1 was efficiently and rapidly depleted from centrioles within two hours of AGB1 addition, but not with cis-AGB1 (Fig. 5b, c). The acute depletion in cycling RPE1 *BromoTag-NEK1* cells resulted in extensions of centriole MTs within two hours, predominantly from mother centrioles (Fig. 5d), indicating that the hyperelongated MTs arise shortly after Nek1 loss. This phenotype was confirmed by electron tomography after Nek1 depletion (Supplementary Fig. 5a). Remarkably, Cep97 and CP110 remained associated with the plus ends of MTs from the early stages of elongation (Fig. 5e–h, Supplementary Fig. 5b), indicating that MT extension occurs without prior removal of the CP110-Cep97 complex.

### CP110-Cep97 and Nek1 work in parallel to restrict centriolar MT extensions from mother and daughter centrioles

Our results showed that 24-hour treatment with AGB1 triggered removal of Nek1 from centrioles in approximately 80% of cells (Fig. 5c). However, centriolar MT elongation was observed in only 35% of cells, and just 5% displayed extensions from both mother and daughter centrioles (Fig. 5d). In *NEK1* KO cells, centriole MT extension occurred in approximately 70% of cells (Fig. 2b). This difference led us to hypothesize that either Nek1 was not sufficiently depleted at some centrioles to trigger MT elongation, or that Nek1 acts in parallel with other factors to inhibit MT extension from centriole tips. Deletion of Nek1 may gradually weaken the inhibitory effects of these cooperative factors, which could explain the more pronounced phenotype in *NEK1* KO cells.

Since we observed very efficient depletion of Nek1 in the degron cell line, we focused on the latter possibility. In addition, *NEK1* KO cells, in which Nek1 is completely absent, also fail to extend centriolar MTs in 100% of the cases (Fig. 2b). Furthermore, mother centrioles in *NEK1* KO cells appear more susceptible to the absence of Nek1 than daughter centrioles (Fig. 3b), suggesting the involvement of additional pathways that suppress distal centriole MT elongation independently of Nek1.

**Fig. 6:**
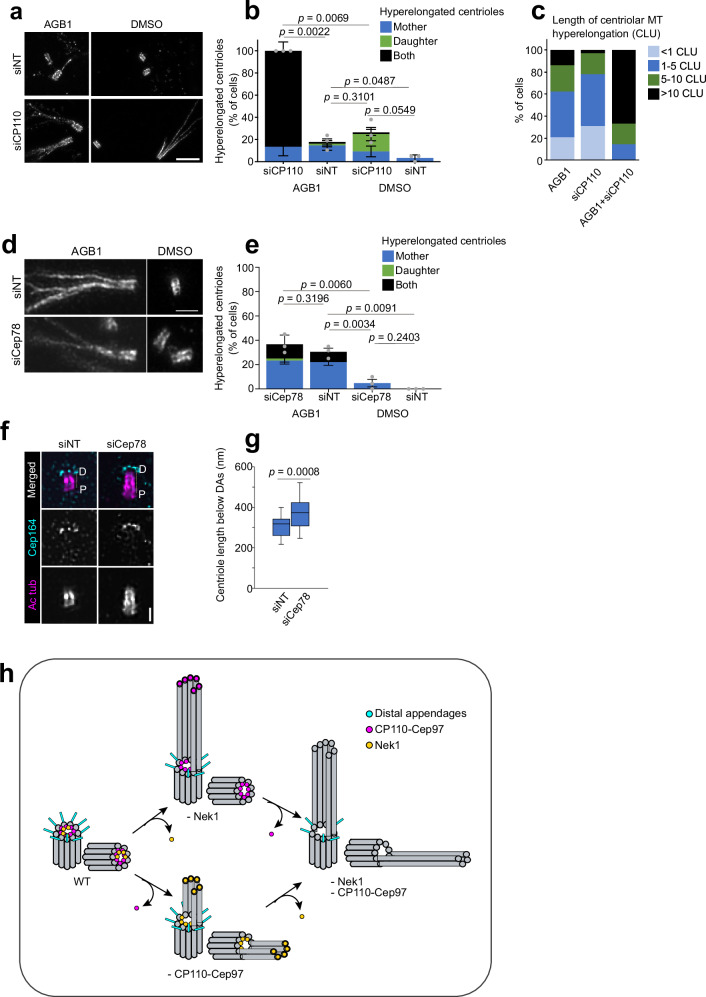
Nek1 acts in parallel with CP110-Cep97 to restrict centriole length at the distal tip. **a** RPE1 *BromoTag-NEK1* cells were treated with non-targeting (siNT) or CP110 siRNA. AGB1 (Nek1 degradation) or DMSO control was added for the last 24 h. The U-ExM shows centrioles stained with acetylated tubulin (Ac tub). Scale bar, 500 nm. **b** Quantification of hyperelongated centrioles from (**a**). Mother centriole: Cep164. Average +/− SD of three independent biological replicates. AGB1+siCP110 *n* = 60, AGB1+siNT *n* = 61, DMSO+siCP110 *n* = 65, DMSO+siNT *n* = 61. Statistics by paired, one-tailed Student’s *t*-test and single data points refer to total elongated centrioles. **c** Estimation of the length of hyperelongated MTs relative to the centriole core length (1 CLU, measured from the proximal end to the DAs) from three (AGB1), four (siCP110), or two (AGB1+siCP110) independent biological replicates. AGB1 *n* = 29, siCP110 *n* = 32, AGB1+siCP110 *n* = 21. **d** RPE1 *BromoTag-NEK1* cells were treated with non-targeting (siNT) or Cep78 siRNA. AGB1 (Nek1 degradation) or DMSO control was added for the last 24 h. The U-ExM shows centrioles stained with acetylated tubulin (Ac tub). Scale bar, 500 nm. **e** Quantification of hyperelongated centrioles in the cells from (**d**). Mother centriole: Cep164. Average +/− SD of three independent biological replicates. AGB1+siCep78 *n* = 60, AGB1+siNT *n* = 59, DMSO+siCep78 *n* = 63, DMSO+siNT *n* = 60. Statistics by paired, one-tailed Student’s *t*-test and single data points refer to total elongated centrioles. **f** Representative U-ExM of mother centrioles stained with acetylated tubulin (Ac tub) and Cep164 of control (siNT) and Cep78 siRNA-depleted cells. Scale bar, 250 nm. **g** Quantification of the length of centrioles in RPE1 *BromoTag-NEK1* cells from (**f**). The distance between the proximal end (P) to the DAs (D, Cep164) was measured as depicted in (**f**). siNT *n* = 22, siCep78 *n* = 31. Median and upper and lower quartiles of six independent biological replicates. Maximum, upper whisker, lower whisker, and minimum values (in nm) are as follows: siNT: 399.29, 399.29, 217.44, 217.44; siCep78: 522.38, 522.38, 246.21, 246.21. Statistics are based on a two-tailed, unpaired Student’s *t*-test. **h** Model depicting the influence of Nek1 and CP110-Cep97 at the centriolar distal tip. CP110-Cep97 restricts the length of mother and daughter centriolar MTs, Nek1 mainly affects the length of mother centrioles. Nek1/CP110-Cep97 co-depletion increases hyperelongation beyond single-depletion levels, indicating that the two pathways synergize to prevent centriole overelongation. Source data are provided as a Source Data file.

We hypothesized that the CP110-Cep97 complex may retain its role in restricting centriole MT length in the absence of Nek1, particularly because it did not become displaced from the distal end of centrioles upon Nek1 depletion. To test this, we performed siRNA-mediated knockdown of CP110 in RPE1 *BromoTag-NEK1* cells together with AGB1 treatment to achieve co-depletion with Nek1. As previously reported^16^, CP110 depletion strongly reduced Cep97 signals at centrioles (Supplementary Fig. 6a, b). U-ExM revealed that depletion of CP110 alone induced centriolar MT extensions in ~20% of cells (siCP110+DMSO, Fig. 6a, b, Supplementary Fig. 6c, d). The presence of hyperelongated centriolar MTs in the absence of CP110 is consistent with previous studies^7,9,17^. Remarkably, co-depletion of Nek1 and CP110 dramatically enhanced this phenotype, both in terms of the percentage of cells displaying elongated centrioles and the length of these extensions (siCP110 + AGB1, Fig. 6a–c). More than 95% of co-depleted cells exhibited very long centriolar MT extensions (>5 CLU, Fig. 6c), with over 80% of these originating from both mother and daughter centrioles (Fig. 6a, b). This indicates that Nek1 may act in a parallel pathway to CP110-Cep97. In support of this hypothesis, Nek1 levels at centrosomes significantly increased upon CP110 depletion, as determined by quantitative immunofluorescence analysis (Supplementary Fig. 6e, f). Interestingly, U-ExM further indicated that Nek1 localization was affected by CP110 depletion. In the absence of CP110, Nek1 remained associated with the distal tip of the majority of non-hyperelongated centrioles (Supplementary Fig. 6g, h, ii and iii). In contrast, in cells with hyperelongated centrioles, Nek1 was absent from the distal tip in about 60% of cases, and in 90% of the centrioles, it was observed along or in close proximity to the elongated centriolar MTs (Supplementary Fig. 6g, h, v and vi). Accumulation of Nek1 in the vicinity of centrioles was also observed in cells without hyperelongated centrioles (Fig. 6g, h, iii, and iv). Together, these findings suggest that CP110 depletion alters Nek1 localization, such that Nek1 is largely retained at the distal tip of non-hyperelongated centrioles but redistributes to the vicinity of centrioles and elongated centriolar MTs upon centriole hyperelongation.

We next wondered whether Cep78 would phenocopy CP110 when co-depleted with Nek1. As reported^38^, single depletion of Cep78 induced centriolar extension, however, asymmetric extension of single MTs appeared in just 5% of the cells (siCep78+DMSO, Fig. 6d, e, Supplementary Fig. 6i, j), and Nek1 localization remained unchanged compared to WT cells (Supplementary Fig. 6k–m). Co-depletion of Cep78 and Nek1 showed the Nek1 phenotype with centriole MT extension in 35% of the cells (siCep78 + AGB1, Fig. 6d, e, compare with Fig. 5d) (Supplementary Fig. 6i, j). Interestingly, we noticed that Cep78-depleted cells displayed elongated centriole cores (Fig. 6f, g), rather than MT elongation beyond DAs, as seen in *NEK1* KO cells. This suggests that Cep78, in contrast to CP110-Cep97 and Nek1, controls centriole core elongation. Thus, our data indicate that the lack of Cep78 does not recapitulate the MT hyperelongation phenotype as observed when CP110-Cep97 or Nek1 is absent.

Together, these findings suggest that Nek1 acts alongside the CP110-Cep97 complex, functioning in parallel and synergistically to ensure timely inhibition of MT hyperextension at distal centriole tips (Fig. 6h).

### Nek1 is removed from the basal body in a Cep78-dependent manner

**Fig. 7:**
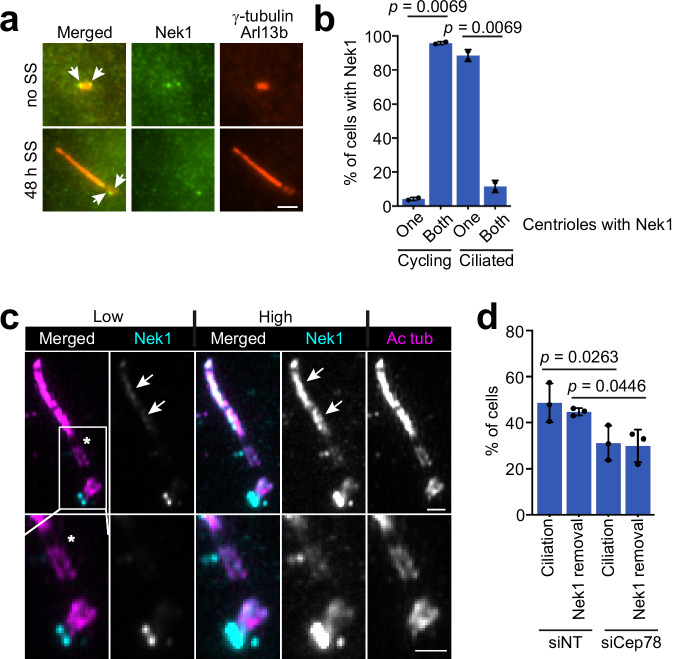
Nek1 is removed from the basal body upon ciliogenesis in a Cep78-dependent manner. **a** Immunofluorescence staining of Nek1 in RPE1 WT cells before (no SS) or after 48 h of serum starvation (48 h SS). The primary cilium was labeled with the cilia membrane marker Arl13b. Centrosomes/centrioles were marked by γ-tubulin. White arrows indicate the two centrioles. Scale bar, 2 μm. **b** Quantification of Nek1 at one or both centrioles in cells from (**a**). The average +/− SD of three independent biological replicates is shown. Cycling cells (no SS) *n* = 426, serum starved cells (48 h SS) *n* = 316. Statistics are based on a paired, one-tailed Student’s *t*-test. **c** U-ExM images of RPE1 WT cells after 48 h of serum starvation. Cells were stained for Nek1 and acetylated tubulin (Ac tub, axoneme, and centrosome marker). Arrows point to Nek1 localization at the ciliary axoneme. The basal body is marked by the asterisk. Enlargements are shown as indicated. Scale bar, 250 nm. **d** RPE1 WT cells were treated with non-targeting (siNT) or Cep78 siRNA for 32 h, and serum starved for 48 h. The percentage of ciliated cells (marked by the cilia membrane marker Arl13b) and the removal of Nek1 from one of the centrioles (marked by γ-tubulin) was quantified by immunofluorescence. The average +/− SD of three independent biological replicates is shown. siNT ciliation, *n* = 327; siNT Nek1 removal, *n* = 317; siCep78 ciliation, *n* = 318; siCep78 Nek1 removal, *n* = 300. Statistics are based on a paired, one-tailed Student’s *t*-test. Source data are provided as a Source Data file.

A prerequisite for primary cilia formation is the removal of the CP110-Cep97 complex from the basal body^16^. Thus, we wondered if Nek1 is also removed from the basal body prior to axoneme extension. To test this, we induced ciliogenesis in RPE1 WT cells by serum starvation for 48 h. Immunofluorescence showed that, whereas Nek1 was present at both centrioles in cycling (non-serum starved) cells, it was absent from the basal body in ciliated cells (Fig. 7a, b). In agreement with previous publications^30,57^, Nek1 localized along the ciliary axoneme (Fig. 7c, arrows).

A key player in CP110-Cep97 removal from the mother centriole is Cep78^21,22^. This protein has been suggested to enhance CP110-Cep97 degradation by recruiting the E3 ubiquitin ligase EDD1-DYRK2-DDB1^VprBP^ to the centrosome^22^. Considering that we identified Cep78 as an interaction partner of Nek1 (Fig. 4f, h), we asked if Cep78 might also be involved in Nek1 removal in serum starved cells. Indeed, Cep78 depletion increased the percentage of cells with Nek1 localizing to both centrioles after 48 h of serum starvation (Fig. 7d). This occurred simultaneously with a decrease in ciliation (Fig. 7d), as reported before^22,58^. We therefore conclude that Nek1 is removed from the basal body in a Cep78-dependent way, which is reminiscent of CP110-Cep97 removal. We suggest that Cep78 might have a promoting function on centriole MT extension by removing Nek1 from the distal end of centrioles, in addition to its role in regulating core centriole length.

## Discussion

Centrioles are conserved MT-based organelles that duplicate once per cell cycle and elongate to a characteristic length that supports centriole’s structure, integrity, and function^2,5^. Despite great progress towards the identification of centriole biogenesis factors, the mechanisms that constrain centriole length within the mitotic cycle remain incompletely understood. Here, we established that the NIMA-family kinase Nek1 is a central player that restricts centriole length in proliferating cells. Our data is consistent with a model in which Nek1 acts synergistically with the well-established CP110-Cep97 capping complex to block centriolar MT hyperelongation on the distal ends of the centrioles (Fig. 6h).

Our data show that *NEK1* KO cells replicate their centrioles and form daughter centrioles, most of which reach an initial length similar to that of WT cells, with a small percentage (about 20%) exhibiting centriolar MT hyperelongation. In contrast, mother centrioles change dramatically. Although core size remains comparable to WT centrioles (about 300 nm), centriolar MTs extend from the distal tip in a large fraction of cells (>60%). Acute Nek1 degradation using a BromoTag-based degron system suggests that mother centrioles elongate faster than daughter centrioles, indicating distinct kinetics of MT growth or the release of blocking mechanisms. Arresting the cell cycle in G_1_ with Palbociclib or by serum deprivation increased the frequency of daughter centriole hyperelongation in the absence of Nek1, indicating that daughters can hyperelongate but do so more slowly or with a delay relative to mothers. Notably, loss of the mother centriole appendage component Cep123 had no effect on mother centriole hyperelongation, suggesting that mother-daughter differences are most likely not dictated by DA components.

The transition zone forms a gate at the ciliary base that regulates trafficking into and out of the cilium and links axonemal MTs to the ciliary membrane, thereby establishing a compartment that enables controlled axoneme extension^59^. In *Drosophila* male germ cells, proper transition zone assembly is required to build the membrane “ciliary cap” that restricts and times axonemal MT growth. Interestingly, when transition zone or ciliary cap formation fails, axonemal MTs elongate prematurely and aberrantly^60^, resembling the phenotype we observed in *NEK1* KO cells. However, in our study using human RPE cell lines, we propose that hyperelongation of centriolar MTs during the mitotic cell cycle in the absence of Nek1 does not result from defective cilia formation or resorption. Several lines of evidence support this view. First, MT extensions occur on both mother and daughter centrioles and form independently of the DA component Cep123, which is essential for axoneme extension^39^. Second, the aberrant extensions span a wide range, from 1 to >100 centriole length units (CLUs), far exceeding the typical cilium length of 7–10 CLUs^61^. Third, extensions begin shortly (within 2 h) after acute Nek1 degradation under conditions not permissive for ciliogenesis (i.e. in serum-fed cells) and, finally, immunofluorescence and electron microscopy analyses detected neither the ciliary marker Arl13b nor a ciliary membrane along these hyperelongated MTs, although isolated vesicles were observed near mother centrioles in both WT and *NEK1* KO or degron cells grown in the presence of serum. Furthermore, analysis of *NEK1*-deficient cells under serum starvation, a condition that induces ciliogenesis, confirmed centriole MT hyperelongation in the absence of a ciliary membrane, consistent with reports that *NEK1* KO cells fail to ciliate^35^. We therefore propose that Nek1 restrains MT extension in cycling cells and enables proper ciliogenesis upon serum starvation. Notably, *NEK1* overexpression blocks cilium formation and destabilizes centrosomes^30,31^, highlighting the importance of maintaining appropriate Nek1 protein levels for centrosome integrity and ciliogenesis.

Nek1 has long been known to associate with human centrosomes, but its substructural distribution has not been determined^30,31^. Our U-ExM analysis now reveals that Nek1 accumulates at the tips of both mother and daughter centrioles throughout the cell cycle. The co-localization of Nek1 with the CP110-Cep97 complex and its interactions with Cep97 observed by two approaches (ReLo and co-immunoprecipitation) indicated that Nek1 and CP110-Cep97 are present in common complexes, most likely at the centriolar tips, where they are perfectly positioned to perform their function in restricting centriolar MT elongation. The absence of Nek1 did not alter CP110-Cep97 localization at the centriolar tip in non-elongated centrioles. Conversely, Nek1 remained associated with the distal tips in the majority (>70%) of non-elongated centrioles lacking CP110-Cep97, indicating that their localization to the centriolar tip is not strictly dependent on one another. However, U-ExM showed that Nek1 accumulated as foci in the vicinity of about 50% of non-elongated centrioles, independently of its distal tip association, most likely explaining the increase in centriolar Nek1 levels detected by conventional immunofluorescence under CP110-depleted conditions. In addition, the distal tip pool of Nek1 was absent in about 20% of non-elongated centrioles, implying that CP110 depletion may directly or indirectly influence Nek1 centriolar binding dynamics. Although we did not detect hyperelongated centriolar MTs in these cases, we cannot exclude the possibility that MTs, whether emanating from centrioles or not, retain Nek1 in the vicinity of centrioles in the absence of CP110-Cep97. Consistent with this notion, Nek1 foci were detected in the vicinity of the majority of hyperelongated centriolar MTs formed upon CP110 depletion, whereas the CP110-Cep97 complex remained associated with centriolar MT plus ends in the absence of Nek1. The fact that the double depletion phenotype was significantly stronger than either single depletion alone indicates that CP110-Cep97 and Nek1 retain activity in the absence of the other, reinforcing the conclusion that CP110-Cep97 and Nek1 are jointly required to control centriolar MT distal tip behavior (Fig. 6h).

MT hyperelongation was detected in almost all centrioles in cells lacking both CP110 and Nek1, including the daughter centrioles. Furthermore, in the context of CP110 depletion, both mother and daughter centrioles became equally hyperelongated, as opposed to mainly mothers in *NEK1*-deficient cells. Based on these differences and the strength of the phenotypes, we propose that the regulation of mother and daughter centrioles differs in proliferating cells. Nek1 and CP110-Cep97 control the length of the mother centriole, with Nek1 playing a more critical role, whereas CP110-Cep97 predominates in controlling the length of the daughter centriole.

Nek1, CP110, and Cep97 are also found at the tips of procentrioles during the elongation phase. It has been reported that CP110-Cep97 supports MT growth at this stage, with centrioles becoming unstable in the absence of the functional complex^62,63^. Nek1 has been shown to regulate MT stability and nuclear-cytoplasmic transport in neurons^32^. However, we did not observe centriole duplication failure or structural instability of daughter centrioles in cells lacking Nek1, which raises questions about the molecular role of Nek1 at the centriolar tip during procentriole formation. Centriole stability is controlled by a number of proteins, including CPAP and Cep350^9,10,38,43^. Therefore, it is possible that Nek1 plays a minor and redundant role in controlling centriolar MT stability during procentriole formation. Interestingly, once centriole elongation is complete, Nek1 and CP110-Cep97 become essential in preventing overly long centrioles, indicating a functional switch. As the majority of daughter centrioles in RPE1 *BromoTag-NEK1* cells upon Nek1 degradation maintain their length in the presence of CP110-Cep97, but not in its absence, it is unlikely that Nek1 itself is involved in the CP110-Cep97 switch. Additional proteins at the distal tip of the centriole may contribute to this process. For example, Cep78 and components of the DISCO complex contribute to centriole length control^12,14,38^. However, unlike in CP110-Cep97- and Nek1-deficient cells, depleting Cep78 promotes elongation of the core centriole. How CP110-Cep97 and Nek1 are regulated during the process of procentriole formation is an interesting question for future studies.

Although centriolar length remains constant throughout the mitotic cell cycle, it changes when the mother centriole becomes the basal body of the cilium. In this scenario, one of the first prerequisites for enabling MT extension at the distal end of the mother centriole is the removal of the CP110-Cep97 complex, given its role in capping and inhibiting MT polymerization^16^. We found that Nek1 exhibits a similar pattern: it is present at the tips of both the mother and daughter centrioles throughout the cell cycle, but is removed from the basal body in ciliated cells. This behavior is consistent with Nek1’s inhibitory role on MT extension in cycling cells, placing it alongside CP110-Cep97 as an inhibitor of ciliogenesis. Interestingly, we discovered that Cep78, a protein that regulates CP110 levels via the EDD-DYRK2-DDB1^VprBP^ E3 ubiquitin ligase complex^21,22^, interacts with Nek1. In the absence of Cep78, Nek1 remains associated with both centrioles upon induction of ciliogenesis, implying that Cep78 may also be involved in the removal of Nek1 from basal bodies. Currently, it is unclear how Nek1 levels decrease at basal bodies. In agreement with previous work^30,57^, we also detected Nek1 along the axoneme, raising the question of whether Nek1 at the mother centriolar tip becomes degraded or relocates with the growing axoneme to the ciliary body.

Nek1 has been implicated in several ciliopathies, including amyotrophic lateral sclerosis (ALS)^64^, polycystic kidney disease (PKD)^65^, and autosomal recessive short-rib polydactyly syndrome, Majewski type^33,34^, as a consequence of mutations that lead to cilia abnormalities. In most *NEK1* mutant cases, the presence of abnormal cilia has been attributed to improper ciliary assembly or disassembly^29,33^. In light of our data, it is sensible to assume that some of the abnormal ciliary phenotypes observed in patient cells or disease-related cell lines lacking Nek1 primarily derive from deregulated centriolar length control. The hyperextension of the centriole in proliferating patient-derived cells^33^ most likely impairs cilia formation, as observed in our ultrastructural analysis. In this context, it would be interesting to understand which disease-related *NEK1* mutations affect the regulation of centriolar length. Identifying the substrates of Nek1 and elucidating the mechanism by which it prevents hyperextension of centriolar MTs will be in future studies an important step for understanding its role in centrosome and cilia biology. Because dysregulation of *NEK1* is implicated in ciliopathies and cancer^24,33,34,64,65^, clarifying its molecular role could help reveal therapeutic entry points. In particular, mapping Nek1’s phosphorylation targets and defining how its activity integrates with other centrosomal kinases may uncover regulatory nodes that can be modulated to restore proper centriole structure or function in disease contexts.

## Methods

### Chemicals and reagents

Unless otherwise stated, all chemicals were purchased from Sigma-Aldrich or Merck. siRNA sequences are listed in Supplementary Table 1, primary and secondary antibodies in Supplementary Table 2 and 3, respectively, and plasmids used in this study in Supplementary Table 4.

### Plasmid construction

All plasmids were cloned using HiFi assembly with the NEB Hifi assembly master mix (NEB) following the manufacturer’s instructions.

For ReLo assay, *NEK1*, *CEP78*, *CEP97*, *CP110*, *C21ORF2*, and *CEP164* were cloned into both pAc5.1 PH-mCherry and pAc5.1-mEGFP^54^. Both vector plasmids were a kind gift from Mandy Jeske, University of Heidelberg, Germany.

For inducible expression of proteins for immunoprecipitation in Hek293T Tet3G cells, *EGFP-CEP78* and *EGFP-CEP97* were amplified from the plasmids used in the ReLo assay and cloned into pESA-SBDonor-TRE3GV-Zeocin^38^ using the primers EGFP-X_ReLo_SB_fwd (5’- TGTCTTATACTTGGATCCATCCCGGATCGGGGTACCATC-3’) and EGFP-X_ReLo_SB_rev (5’- CTCCCCTACCCGGTAGAATTTCAATGGTGATGGTGATGATGAC-3’). *mEGFP-CP110* was cloned from the ReLo plasmid into pRetroX-Tet3G containing 2x*FLAG*.

### Cell culture and transfection

Cells used in this study were hTERT-immortalized RPE1 (ATCC CRL-4000), ARPE-19^35^, ARPE-19 *NEK1* KO and *NEK1* KO stably expressing either *NEK1* WT (also referred to as *NEK1* KO + WT) or *NEK1* D146A (also referred to as *NEK1* KO+Nek1-KD)^35^, RPE1 *BromoTag-NEK1*, RPE1 *TP53* KO (Kind gift from Bryan Tsou^66^), RPE1 *CEP83* KO (Laboratory stock), RPE1 *TP53* KO *CEP350* KO (Karasu et al., 2022^38^), RPE1 *OFD2* KO (Viol et al., 2020^37^), Hek293T (ATCC CRL-3216) stably expressing Tet3G, and *Drosophila* S2R+ (kind gift from Mandy Jeske, University of Heidelberg, Germany).

RPE1 and ARPE-19 cells were cultured in DMEM/F12 (Sigma-Aldrich # D6421) supplemented with 10% FBS (RPE1 and ARPE-19 WT) or 20% FBS (*NEK1* and *C21ORF2* KO cell lines), sodium bicarbonate, and 1% L-glutamine. For ARPE-19 *NEK1* KO+Nek1-KD, 2 μg/ml puromycin was added for continuous selection. Degradation of Nek1 in RPE1 *BromoTag-NEK1* cells was induced by the addition of 600 nM AGB1 or cis-AGB1 (BioTechne). Hek293T cells were cultured in DMEM high glucose medium (Sigma/Merck # D6429) supplemented with 10% FBS. All mammalian cell lines were grown at 37 °C and 5% CO_2_. S2R+ cells were cultured in Schneider’s *Drosophila* medium (Gibco #21720024) supplemented with 10% FBS, and antibiotic/antimycotic (Gibco) at 26 °C.

For transfection of S2R+ cells, 300,000 cells were seeded to an 8-well 1.5 μ-slide (Ibidi) and transfected with 300 ng total plasmid and jetOPTIMUS® transfection reagent (Avantor) with jetOPTIMUS® buffer (Avantor).

Transient transfection of Hek293T cells was achieved by growing the cells to 60–70% confluency in 10 cm plates and transfecting them with 10 μg of each plasmid using polyethylenimine (PEI, 25 kDa) reagent. A ratio of 1 to 2.5 (Plasmid DNA to PEI) was used in all transfections.

The medium was changed 16 h after transfection. For the inducible Tet-promoter, 1 μg/ml doxycycline was added to the medium. Cells were incubated for 48 h total time before analysis.

All cell lines were regularly tested for mycoplasma using the Mycoplasma Check service (Eurofins Genomics).

### Generation of RPE1 *BromoTag-NEK1* cells

For transfection, RPE1 cells were seeded at a density of 1.5 × 10^6^ cells per 10 cm dish and transfected with PEI using 1.5 μg each of the plasmids nickase pair sense-2 (DU57081) and nickase pair antisense-2 (DU57089), together with 3 μg of the donor plasmid (DU74281). After 24 h, the medium was replaced with complete medium supplemented with puromycin at a final concentration of 25 μg/ml. Cells were maintained under selection for four days to enrich for positively transfected populations.

Surviving cells were transferred to a 15 cm dish and allowed to recover in complete medium for 24 h before re-transfection with double the initial amounts of each plasmid. Fresh medium was added 24 h post-transfection, and cells were incubated for an additional two days before proceeding to fluorescence-activated cell sorting (FACS) to isolate GFP-positive cells.

Cells were prepared for FACS by incubating plates with Trypsin-EDTA (Gibco, Fisher Scientific) for at least 5 minutes to detach cells. The cell suspension was resuspended in complete medium, centrifuged at 300 x *g* for 5 min, washed with phosphate-buffered saline (PBS, Gibco), and resuspended in media containing 1% fetal bovine serum (FBS, Gibco) at a final concentration of 5 × 10^6^ cells/ml.

Sorting was performed at the Flow Cytometry and Cell Sorting Facility at the University of Dundee using an MA900 cell sorter (Sony Biotechnology). GFP-positive RPE1 cells were collected into individual wells of three 96-well plates, each containing 200 μl of preconditioned medium supplemented with 20% FBS. Immediately after sorting, plates were centrifuged at 100 x *g* for 1 min and incubated under standard culture conditions (37 °C, 5% CO₂, 95% humidity).

After 12 days of incubation, a total of 72 colonies were expanded. Of these, 54 surviving colonies were subjected to WB analysis to verify the presence of Bromo-tagged *NEK1* alleles. For this purpose, cells were lysed using 50 mM Tris (pH 7.5), 150 mM NaCl, 0.27 M Sucrose, 1% Triton X-100, and 0.5% NP-40 substitute, freshly supplemented with protease inhibitors (Complete™ Mini EDTA-free, Roche), Universal nuclease (50 U/ml, Pierce), and β -Mercaptoethanol. Protein lysates were quantified using the Bradford assay. Subsequently, lysates were mixed with LDS sample buffer (NUPAGE, ThermoScientific) and 5% (v/v) DTT and heated at 95 °C for 5 min.

Protein samples were resolved by SDS-PAGE on 3–8% Tris-Acetate gels (NUPAGE Novex, ThermoFisher) at 100 V for 1 h 20 min. WBs were probed using a sheep anti-Nek1 antibody raised by the MRC PPU Reagents and Services team at the University of Dundee (SA354). Bromo-tagged Nek1 was identified by a characteristic 15 kDa band shift relative to wild-type Nek1. Clones displaying only the expected band shift were classified as homozygous candidates and selected for genomic integration verification of the Bromo-Tag in the *NEK1* locus via junction PCR.

Genomic DNA from 5 positive candidates showing the Nek1 band shift was extracted using the DNeasy Blood & Tissue Kit (Qiagen) according to the manufacturer’s instructions. To check for integration of the Bromo-Tag in the *NEK1* locus, PCR reactions were performed with 300 ng of genomic DNA, the PrimeStar GXL polymerase kit (Takara) plus the following primer combinations: NTer F (5’-AGTGTTCTTTAACTACTACCCTTTATTGCTTCTC-3’) and NTer RW (5’-GCATGATATCAGTATTGTCCTTGAATCAAGC-3’) (flanking homology regions), NTer F and GFP RW (5’-CTCGTTGGGGTCTTTGCTCAGG-3’) (targeting GFP), or BTag F (5’-GCATCCTCAAGGAGATGTTTGCC-3’) (targeting BromoTag) and NTer RW. Amplified bands of predicted sizes confirmed successful integration, and positive clones were further characterized.

### RNA interference

For siRNA depletion of Cep123, 1.5 × 10^5^ cells were seeded on 10 mm coverslips (ThermoFisherScientific) in a 6-well plate and reverse-transfected with 50 nM siRNA using Lipofectamine RNAiMAX (ThermoFisherScientific) for 48 h. For depletion of CP110, 0.9 × 10^5^ cells per well were seeded to a 6-well plate and reverse-transfected with 20 nM siCP110 for 32–48 h. To deplete Cep78, 0.9 × 10^5^ cells per well were seeded to a 6-well plate and reverse-transfected with or 50 nM siCep78 or Cep78_1. Cells were analyzed after 32–48 h.

### Cell lysis and Western blotting

Cells were grown to 80–90% confluency in a 6-well plate. After washing thrice in ice-cold PBS, the cells were scraped, collected in PBS, and centrifuged at 800 x g for 3 min at RT. The pellet was resuspended in RIPA buffer (50 mM Tris-HCl (Roth), pH 8.0; 150 mM NaCl (Labochem® international); 1% NP-40 (Fluka); 0.1% SDS (Roth); 0.5% sodium deoxycholate) supplemented with Complete EDTA-free protease inhibitor cocktail (Roche). The lysis was performed on ice for 30 min. If necessary, 1:1000 Benzonase (250 Units/μl, Millipore) was added for 10 min on ice. The protein concentration was determined using the Pierce^TM^ BCA Protein Assay Kit (ThermoScientific) following the manufacturer’s instructions. For blotting, the lysate was supplemented with 4x loading buffer (200 mM Tris-HCl, pH 6.8; 8% SDS; 0.4% bromophenol blue (Electran®); 40% glycerol (Labochem® international); 400 mM β-mercaptoethanol) and heated to 65 °C for 15 min. Then, the samples were run on an 8% SDS-Page gel and transferred to a PVDF membrane (Amersham^TM^ Hybond^TM^). The membranes were blocked in 5% milk/0.02% Tween-20 (Roth)/PBS for 30 min at room temperature (RT) and probed with primary antibody in 3% milk/0.02% Tween-20/PBS overnight at 4 °C. Secondary antibodies conjugated to HRP were incubated for 1 h at RT. The blots were visualized on ImageQuant800 (Cytiva Amersham) using the Cytiva Amersham ImageQuant800 (v. 2.0.0) software.

### Co-Immunoprecipitation

Co-IP was performed following the protocol from Gregorczyk *et al*. (2023)^35^. In brief, Hek293T cells were transfected with *3xFLAG-NEK1* using the plasmid pcDNA FRT TO *3xFLAG-NEK1*^35^ and the protein of interest. Expression was induced with 1 μg/ml doxycycline for 36-48 h. Cells were harvested at 100% confluency with 1x ice-cold PBS. Then cells were lysed with lysis buffer (50 mM Tris-HCl, pH = 7.4; 150 mM NaCl; 270 mM sucrose; 1 mM EGTA, pH 8.0 and 1% (v/v) Triton X-100) supplemented with 0.5 U/ml Benzonase Nuclease (Millipore), complete EDTA-free protease inhibitor (Roche), phosphatase inhibitor cocktail 2 (ThermoScientific), 1 mM sodium orthovanadate, 1 mM Pefabloc^TM^, 1 mM Benzamidine, 5 mM sodium pyrophosphate decahydrate, 10 mM β-glycerol phosphate disodium salt pentahydrate and 50 mM sodium fluoride). The lysate was incubated on ice for 20 min and cleared by centrifugation at 14,000 *g* for 15 min at 4 °C. The GFP-Trap beads were equilibrated with the lysis buffer by washing the beads with the buffer 3 times. An equal amount of protein lysate for each condition was incubated with the GFP-Trap beads on a rotating wheel for 2 h. The beads were washed 5 times with ice-cold lysis buffer, resuspended in SDS Laemmli sample buffer, and analyzed by SDS-Page and Western blot.

### ReLo assay

The ReLo assay was performed according to Salgania et al. (2024)^54^. Equal amounts of the mEGFP and mCherry plasmids were transfected into SR2+ cells using jetOPTIMUS (Polyplus Transfection). Transfected cells were cultured for 24 h before live-cell microscopy analysis at a Nikon AX confocal microscope using a Plan Apo λ 60x Oil Ph3 DM oil Nikon objective, Iris 9 Camera (Photometrics), and NIS-Elements AR software version 5.42.06 (Nikon).

### Immunofluorescence microscopy

For immunofluorescence, cells were grown on 10 mm coverslips (Standard #1.5, ThermoScientific) in 24-well plates. Cells were washed once with PBS. Methanol fixation was done for 5 min at −20 °C. For PFA+methanol fixation, cells were first fixed in 3% PFA (Acros Organics)/PBS at RT for 3 min. Following removal of the PFA, cells were permeabilized in methanol at −20 °C for 5 min. Cells were blocked for 30 min in 3% BSA/0.075% Triton X-100/PBS. Primary and secondary antibody dilutions were prepared in blocking buffer. Primary antibodies were incubated for 1 h, secondary antibodies for 30 min at RT. 50 ng/ml DAPI was added to the secondary antibody dilution. Finally, the coverslips were mounted in mounting medium (20% (w/v) Mowiol® 4-88 (Merck Millipore); 50% (w/v) glycerol; 2.4% (v/v) Dabco® 33-LV; 0.2 M Tris, pH 8.5) and dried overnight at RT.

Z-stack images (spacing 0.3 μm) were taken on a Nikon Eclipse Ti2 microscope using a Plan Apo λ 60x Oil Ph3 DM oil Nikon objective, Iris 9 Camera (Photometrics), and NIS-Elements AR software version 5.42.06 (Nikon). Figures in Supplementary Fig. 1e were taken at the DeltaVision RT system (Applied Precision) with an Olympus IX71 microscope equipped with 60×/1.42 and 100×/1.40 oil objective lenses and the softWoRxv6.1.1 Release 5 (AppliedPrecision, GE) software. Images show the maximum Z projection or best focal plane.

### Staining of S-phase cells with EdU

EdU (5-Ethinyl-2’-desoxyuridin) staining was performed using the Click-iT^TM^ Plus EdU Alexa FluorTM 555 Imaging Kit (Invitrogen #C10638) following the manufacturer’s protocol with some modifications. In brief, cells were incubated with 10 μM EdU for 20 min prior to methanol fixation. After blocking in 3% BSA/0.075% Triton X-100/PBS for 30 min, the Click-iT® Plus reaction cocktail was prepared according to the manufacturer’s instructions, and coverslips were incubated with 20 μl of this cocktail for 20 min for EdU detection. After washing three times with PBS, primary and secondary antibody staining were performed as described above.

### Ultrastructure expansion microscopy (U-ExM)

For U-ExM, cells were cultured on coverslips as described above. The procedure was modified from Gambarotto et al. (2019)^67^. In brief, cells were washed once with PBS, pre-fixed with ice-cold methanol and/or extracted using CSK buffer (10 mM potassium PIPES buffer pH 7.0, 33.3 mM Sucrose (AnalaR®), 100 mM NaCl, 0.33 mM MgCl_2_, 10 mM EGTA pH 8.0, 0.5% Triton X-100). After washing with PBS, cells were fixed in 0.7% formaldehyde/1% acrylamide (Bio-Rad) in PBS for 4–5 h at 37 °C and incubated 5 min on ice and 1 h at 37 °C in MS solution consisting of 21.1% sodium acrylate (AK Scientific), 11.1% acrylamide, 0.22% *N, N*′-methylenbisacrylamide (Roth) in PBS, freshly supplemented with 0.5% *N, N, N*′*, N*′*-*Tetramethylethylenediamine (TEMED, Roth) and 0.5% ammonium persulfate (APS). For denaturation, cells were first incubated in denaturation buffer (5.71% SDS; 200 mM NaCl; 0.6% Tris, pH 9.0) at RT for 15 min, then detached from the coverslips and incubated in denaturation buffer at 95 °C for 30 min. The gels were expanded in ddH_2_O for 1 h under shaking, changing the water every 20 min, and the expansion factor was determined. Before antibody staining, gels were shrunk by incubation in PBS for 20 min at room temperature, exchanging the PBS three times during this period, and cut into quarters. Primary antibodies were diluted in 1% BSA/PBS and incubated overnight in a 24-well plate at 37 °C under shaking. The gels were washed three times for 10 min in 0.1% Tween20/PBS on a rocking shaker, then incubated with the secondary antibodies and DAPI for 2.5 h at 37 °C under shaking. After another three washes with 0.1% Tween20/PBS, gels were expanded for 3 × 20 min in ddH_2_O as before, and placed on a dish (MaTek) coated with poly-l-lysine for imaging.

Z-stack images (spacing 0.12–0.3 μm) were taken on the Nikon Eclipse Ti2 microscope using a Plan Apo λ 100x Oil Ph3 DM oil Nikon objective, Iris 9 Camera (Photometrics), and NIS-Elements AR software version 5.42.06 (Nikon), or on the Nikon AX confocal microscope with a Plan Apo λ 100x Oil Ph3 DM oil Nikon objective, and NIS-Elements AR software version 5.42.06 (Nikon), or on the Leica TCS SP8 STED 3X microscope with FALCON FLIM with the HCX PL APO 63x/1.40 Oil CS2 or HC PL APO CS2 100x/1.40 Oil objective and the Leica Falcon LASX Flim v.3.5.7 (Leica Application Suite X) software (ZMBH imaging facility, Heidelberg, Germany). Images shown were taken using the following microscopes: Nikon AX for Figs. 1c, 2a (overview), Fig. 3a (Sas6 and Cep135), c, Figs. 5e, g, 6a, d, f, 7c, Supplementary Figs. 3c, g, 6a, j; Eclipse Ti2 for Fig. 1e, Supplementary Fig. 2d; Leica SP8 for Figs. 1a, 2a (detailed views), f, g, i, Figs. 3a, d, f, h, 4a–c, Supplementary Fig. 1a–c, Supplementary Fig. 3e, i, k, m, o, Supplementary Fig. 4a-d.

3D deconvolution of the images taken from the Nikon microscope was performed on NIS-Elements AR Analysis 5.30.06 (Nikon) using the Richardson-Lucy algorithm. Images taken on the Leica microscope were deconvolved with Huygens’ Deconvolution software v18.10.0p7 (SVI Inc.) using the standard 3D-deconvolution protocol. Figures show the maximum Z projection of the stacks containing the centrioles or the best focal plane. Scale bars are adjusted to an expansion factor of four.

### Image analysis and processing

Microscope images were analyzed using ImageJ2 version 2.16.0/1.54p^68^. Presence or absence of fluorescent signal was determined at the centrosome/basal body focal plane. Fluorescence intensity was measured from the maximum projection of 17 Z stacks or the best focal plane (Supplementary Fig. 2i) using a macro adapted from Viol et al.^37^. The mean signal intensity was measured in a square of 8 μm^2^ around the centrosome as stated in the figure legends. Background of the same channel was measured in a square of 32.1 μm^2^ around the same central point and subtracted from the signal. Any negative values were set to zero. Signal intensities were normalized to the average signal of the control of each replicate. The signal intensity along the centrosome in U-ExM was measured in ImageJ2^68^ using the PlotProfile. The signal intensities of each protein were normalized to the average signal intensity of the same protein across the entire distance. Apart from brightness and contrast adjustment, no further modifications were made.

For measuring protein distribution at centrioles or centriole length in ImageJ2^68^, the signal length was normalized to the expansion factor of the respective gel or to the core centriole length. Where protein distribution along the centriole was measured, 150–200 nm of hyperelongating centriolar MTs above the centriole were included for *NEK1* KO cells. Unless otherwise stated, mitotic cells were not considered for quantification.

All figures were assembled using Inkscape (Version 1.4) and Adobe Illustrator 3.30.

### Statistical analysis

Statistics were performed on the average values of each biological replicate in bar graphs and for single values in boxplots. Paired, one-tailed Student’s t-tests were applied where presence or absence of a protein or centriolar hyperelongations was investigated (Figs. 2b, 3i, j, 5c, 6 b, e, 7b, d, Supplementary Figs. 1d, 2f, g), two-tailed, unpaired Student’s t-tests assuming unequal variances were used in the boxplots and the rescue experiment in Supplementary Figs. 2a, b, 3b, 6k. Statistics in Fig. 1f, g by two-tailed, unpaired Student’s t-test refer to the distance of the proximal end of the protein signal from the proximal end of the centriole. In Figs. 4d, 5f, h, Supplementary Fig. 3f, h, j, l, n, p, statistics on protein localization at the centriole wall by two-tailed, unpaired Student’s t-test refer to the total signal length of the respective protein. The sample size and numbers of biological replicates are indicated in the figure legends. Bar graphs show the average value +/- SD. Boxplots represent the upper and lower quartiles. The line indicates the median. Any values outside the range of 1.5 times the interquartile range were considered as outliers and are included in the boxplots.

### Electron microscopy

Cells were seeded on coverslips and cultured at 37 °C and 5% CO_2_ until they reached a confluency of approximately 60–80%. Cells were rinsed with PBS and pre-fixed with a gentle warm-up mixture of 2.5% glutaraldehyde/1.6% PFA/2% sucrose in 50 mM cacodylate buffer for 30 min at room temperature. After 3 times rinsing of the cells with cacodylate buffer, cells were post-fixed with 2% OsO_4_ for approximately 45 minutes on ice, in darkness. Right after, the cells were washed with distilled water (dH_2_O) and incubated overnight at 4 °C in aqueous 0.5% uranyl acetate. On the following day, coverslips were rinsed with dH_2_O and subsequently stepwise dehydrated with ethanol, starting at 40%, ending up to 100% ethanol. Coverslips were immediately placed on capsules filled with Spurr resin and polymerized at 60 °C for approximately two days. In resin-embedded cells were serial-sectioned using a Reichert Ultracut S Microtome (Leica Instruments) to a thickness of approximately 80 nm. Post-staining was performed with 3% uranyl acetate in aqueous solution and lead citrate. Serial sections were imaged at a Jeol JE-1400 (Jeol Ltd.), operating at 80 kV, equipped with a 4k x 4k digital camera (F416, TVIPS). Micrographs were adjusted in brightness and contrast by applying ImageJ 1.54p software^68^.

For tomography, RPE1 *BromoTag-NEK1* cells were treated with AGB1 or cis-AGB1 for 24 h. Tilt series from approximately 250 thick sections were acquired on an electron microscope (FEI Tecnai F20 TEM) operated at 200 kV and equipped with a field emission gun and bottom-mounted 4 K camera. The grid was placed in a high-tilt holder, and images were recorded in single-axis tilt series over a −60° to 60° tilt range (1° increment). SerialEM software version 4.1 was used to collect data for tomography and further processed in eTomo version 23.0.1 (0) to generate tomograms and IMOD software 4.0.29 for creating 3D reconstructions and modeling.

### Reporting summary

Further information on research design is available in the Nature Portfolio Reporting Summary linked to this article.

## Supplementary information

Supplementary Information

Reporting Summary

Transparent Peer Review file

## Source data

Source data

## Data Availability

All data generated in this study are provided in the Supplementary Information/Source Data file. Source data supporting the figures are provided with the paper. Source data are provided with this paper.
