# Supplementary material for: Nek1 defines a branch of centriolar microtubule length control parallel to CP110-Cep97
Source: Nat Commun. 2026 Jun 9;17:7330. doi: 10.1038/s41467-026-73560-9 (PMC13402741; doi:10.1038/s41467-026-73560-9)

Nek1 Defines a Branch of Centriolar Microtubule Length Control Parallel to CP110-Cep97

J. M. S. Streubel [#], O. R. Karasu [#], I. M. Munoz, A. Neuner, E. N. Numanoglu, T. J. Macartney, E. Schiebel, J. Rouse, G. Pereira**

[#] equal contribution

* co-corresponding authors (gislene.pereira@cos.uni-heidelberg.de; g.pereira@dkfz.de; j.rouse@dundee.ac.uk)

Supplementary Figures

Supplementary Figure 1

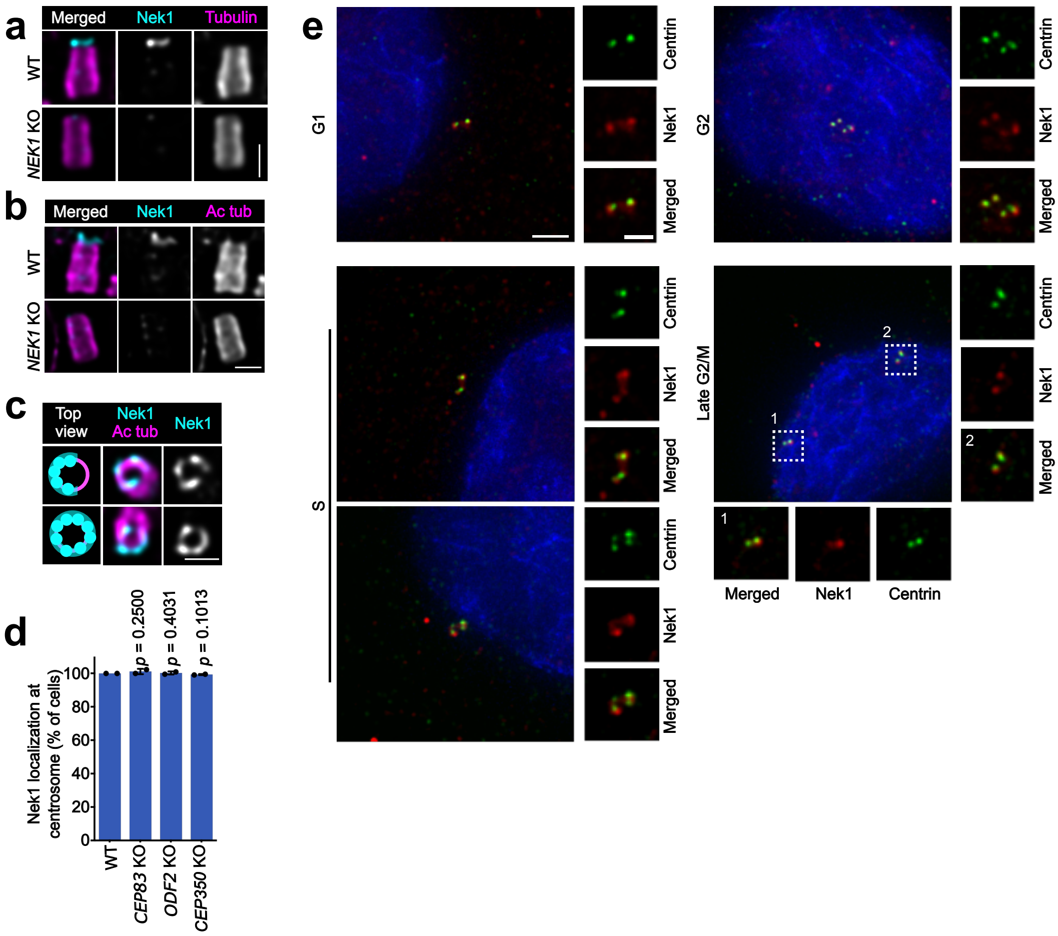

Supplementary Figure 1. Nek1 localization is independent of cell cycle stage and appendage proteins.

a) U-ExM image showing centrioles (α-tubulin) of ARPE-19 WT and *NEK1* KO cells stained for Nek1. Scale bar, 250 nm. Representative images from two independent biological replicates.

b) U-ExM image showing centrioles (acetylated tubulin, Ac tub) of ARPE-19 WT and *NEK1* KO cells stained for Nek1 using a commercially available anti-Nek1 antibody (Abcam #ab229489). Scale bar, 250 nm. Representative images from 25 cells (WT) and 8 cells (*NEK1* KO).

c) Top view U-ExM images of centrioles (acetylated tubulin, Ac tub) with Nek1 stained with a commercially available antibody (Abcam #ab229489). Scale bar, 250 nm. Representative images from 21 cells.

d) Quantification of presence or absence of Nek1 at the centrosome (γ-tubulin) by immunofluorescence staining in RPE1 *TP53* KO, RPE1 WT, *ODF2* KO, *CEP83* KO, or *TP53* KO *CEP350* KO cells. Average +/- SD from two independent biological replicates. WT/*TP53* KO n=890, *CEP83* KO n=306, *ODF2* KO n=446, *CEP350* KO n=324. Statistics are based on paired, one-tailed Student's t-test.

e) Immunofluorescence images of Nek1 at the centrosome (Centrin) in different cell cycle stages. Enlargements indicate the centrosomal area. Scale bar, 5 μm; inlets, 2 μm. Representative images from 20 cells. Source data are provided as a Source Data file.

Supplementary Figure 2

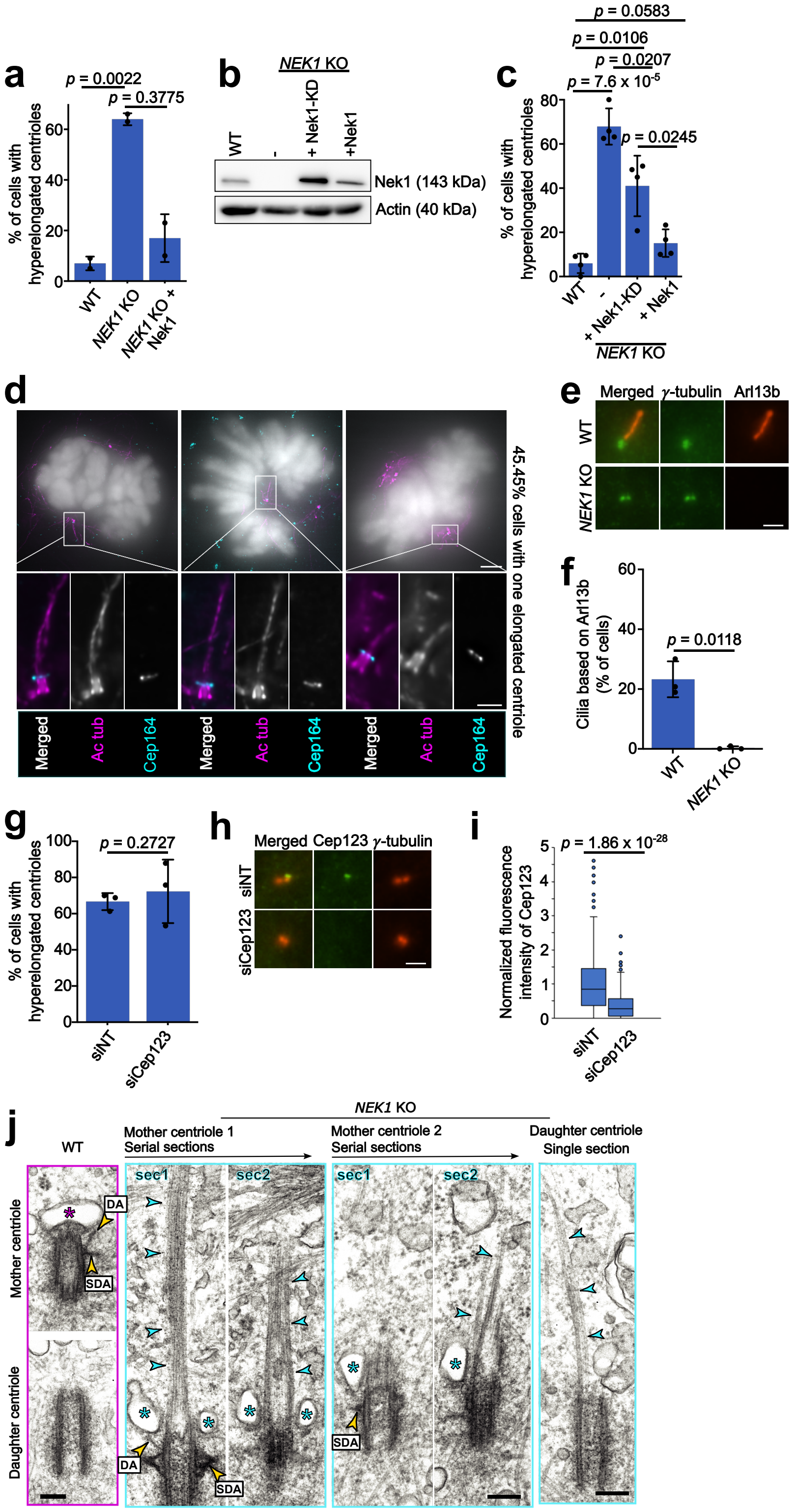

Supplementary Figure 2. Characterization of centriole hyperelongations.

a) Quantification of the percentage of hyperelongated centrioles in ARPE-19 WT, *NEK1* KO, or *NEK1* KO cells expressing full length *NEK1* (+Nek1). Hyperelongated centrioles were determined

by U-ExM based on acetylated tubulin. Average +/- SD from two independent biological replicates. WT n=60, *NEK1* KO n=73, *NEK1* KO+WT n=60. Statistics are based on two-tailed, unpaired Student's t-test.

b) Western blot showing Nek1 expression levels in ARPE-19 WT, *NEK1* KO and *NEK1* KO cells expressing a kinase-dead variant of *NEK1* (D146A, +Nek1-KD) or full length *NEK1* (+WT). Actin is shown as a loading control. Representative blot from three independent biological replicates.

c) Quantification of the percentage of hyperelongated centrioles in ARPE-19 WT, *NEK1* KO, or *NEK1* KO cells expressing kinase-dead (D146A, +Nek1-KD) or full length *NEK1* (+WT*)*. Hyperelongated centrioles were determined by U-ExM based on acetylated tubulin. Average +/- SD from four independent biological replicates. WT n=101, *NEK1* KO n=112, *NEK1* KO+Nek1-KD n=100, *NEK1* KO+WT n=99. Statistics are based on two-tailed, unpaired Student's t-test.

d) U-ExM of ARPE-19 *NEK1* KO cells in different phases of mitosis stained for acetylated tubulin (Ac tub) and Cep164 as a mother centriole marker. Enlargements of the centrosomal area are shown. Scale bar, 2.5 μm; enlargement, 500 nm. Cells from three independent biological replicates were quantified. n=11.

e) Ciliogenesis in cycling ARPE-19 and *NEK1* KO cells was analyzed by immunofluorescence staining for γ-tubulin (centrosome marker) and Arl13b (ciliary membrane marker). Scale bar, 5 μm.

f) Quantification of **(e)** from three independent biological replicates (average +/- SD). WT n=315, *NEK1* KO n=333. Statistics are based on paired, one-tailed Student's t-test.

g) Quantification of the percentage of ARPE-19 *NEK1* KO cells displaying hyperelongated centriolar MTs following control (siNT) or Cep123 siRNA depletion. The quantification was performed by U-ExM based on acetylated tubulin from three independent biological replicates (average +/- SD). siNT n=93, siCep123 n=96. Statistics are based on paired, one-tailed Student's t-test.

h-i) Control of Cep123 depletion. Representative images show Cep123 levels at centrosomes (stained with γ-tubulin) in ARPE-19 *NEK1* KO cells treated with non-targeting (siNT) or Cep123 siRNAi **(h)**. Scale bar, 2 μm. Normalized fluorescence intensity quantifications of Cep123 levels at centrosomes **(i)** from three independent biological replicates. siNT n=304, siCep123 n=303. The boxplot shows the median and upper and lower quartiles of three independent biological replicates. Statistics are based on two-tailed, unpaired Student's t-test. Maximum, upper whisker, median, lower whisker, minimum values are as follows: siNT: 4.61, 3.24, 0.85, 0.00, 0.00; siCep123:2.40, 1.44, 0.27, 0.00, 0.00.

j) Electron micrographs of centrioles in ARPE-19 WT (magenta) and *NEK1* KO (cyan) cells serum starved for 48 h. Cyan arrowheads indicate hyperelongated MTs, asterisks indicate vesicles close to the base of centriole hyperelongations, and yellow arrowheads point to distal appendages (DA) or subdistal appendages (SDA) as indicated. Sec 1 and sec 2 represent consecutive sections through the same centriole. Scale bar, 150 nm. Representative images from 11 (WT) or 7 (*NEK1* KO) cells are shown. Source data are provided as a Source Data file.

Supplementary Figure 3

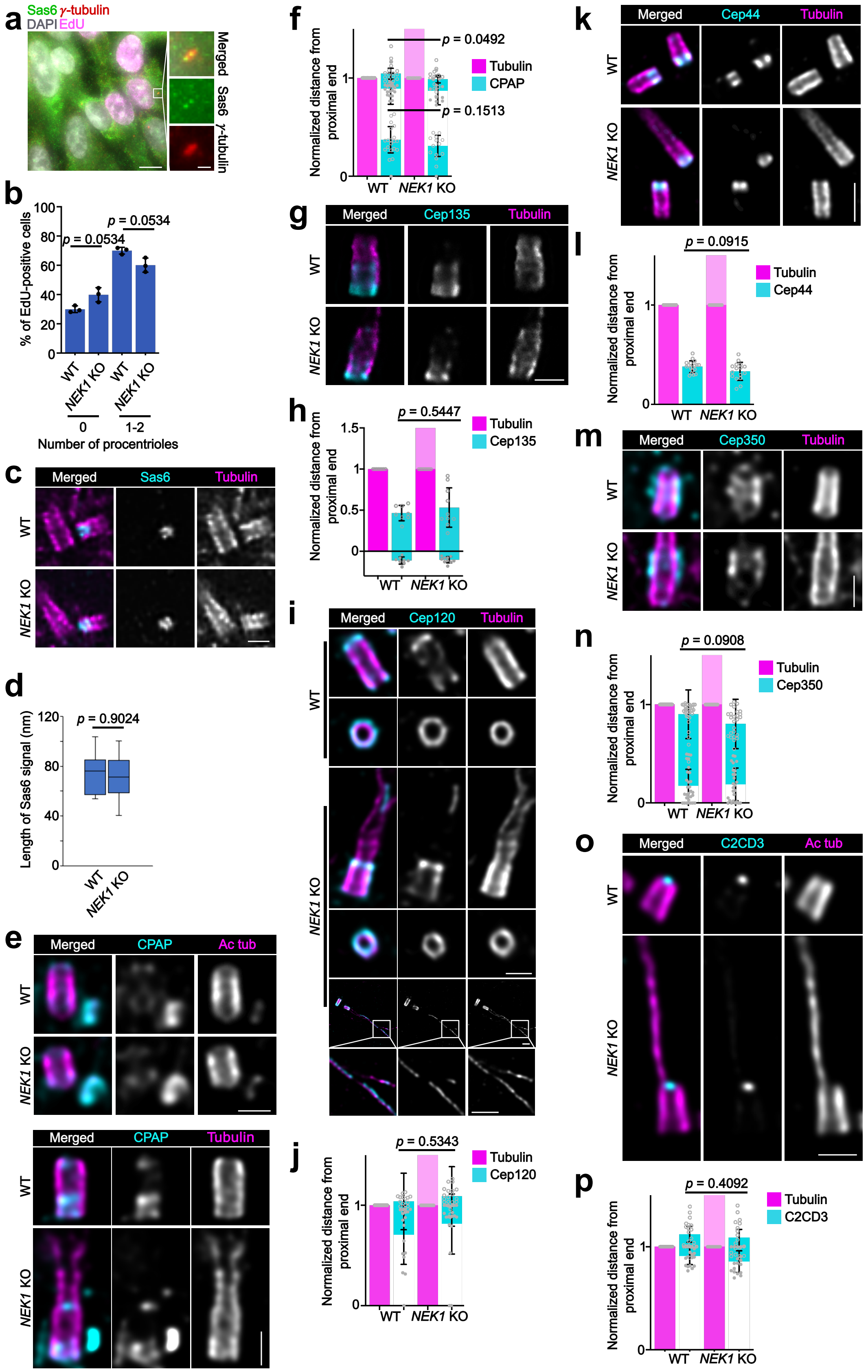

Supplementary Figure 3. Localization of centriolar proximal proteins in ARPE-19 *NEK1* KO cells.

a) Immunofluorescence images of ARPE-19 *NEK1* KO cells stained with DAPI (nucleus), EdU (S phase marker), γ-tubulin (centrosome marker), and Sas6 (procentriole marker). Scale bar, overview, 10 μm; enlargement, 1 μm.

b) Quantification of the number of procentrioles per centrosome in EdU positive ARPE-19 WT and *NEK1* KO cells from **(a)**. The average +/- SD of three biological replicates is shown. WT n=133, *NEK1* KO n=143. Statistics are based on two-tailed, unpaired Student's t-test.

c) Representative images of U-ExM showing the centrosomes of ARPE-19 WT and *NEK1* KO cells stained for α-tubulin (centriole marker) and Sas6. Scale bar, 250 nm.

d) Quantification of the length of Sas6 signal at procentrioles from **(c)**. Median, upper and lower quartiles of two independent biological replicates are shown. WT n=7, *NEK1* KO n=14. Statistics are based on two-tailed, unpaired Student's t-test. Maximum, upper whisker, median, lower whisker, minimum values (in nm) are as follows: WT: 103.66, 103.66, 76.01, 53.94, 53.94; *NEK1* KO: 100.17, 100.17, 71.42, 57.22, 57.22.

e) Representative images of U-ExM showing the centrosomes of ARPE-19 WT and *NEK1* KO cells stained for α-tubulin and CPAP. Scale bar, 250 nm.

f) Quantification of the length of CPAP signal at the centrioles from **(e)**. The signal length was normalized to the length of the respective core centriole. For *NEK1* KO cells, only hyperelongated centrioles were quantified. The lower and the upper signal boundaries are indicated by filled and open circles, respectively. Average +/- SD of two independent biological replicates. WT n=19, *NEK1* KO n=13. Statistics are based on two-tailed, unpaired Student's t-test.

g) Representative images of U-ExM showing the centrosomes of ARPE-19 WT and *NEK1* KO cells stained for α-tubulin and Cep135. Scale bar, 250 nm.

h) Quantification of the length of Cep135 signal at the proximal end of centrioles from **(g)**. The signal length was normalized to the length of the respective core centriole. For *NEK1* KO cells, only hyperelongated centrioles were quantified. The lower and the upper signal boundaries are indicated by filled and open circles, respectively. Average +/- SD of two independent biological replicates. WT n=8, *NEK1* KO n=10. Statistics are based on two-tailed, unpaired Student's t-test.

i) Representative images of U-ExM showing the centrosomes of ARPE-19 WT and *NEK1* KO cells stained for α-tubulin and Cep120. The two lower panels (overview and enlargement) show Cep120 localization to MTs. Scale bar, 250 nm; enlargement, 500 nm.

j) Quantification of the length of Cep120 signal at centriolar distal tips from **(i)**. Only the location of the most prominent distal pool was quantified. The signal length was normalized to the length of the respective core centriole. For *NEK1* KO cells, only hyperelongated centrioles were quantified. The lower and the upper signal boundaries are indicated by filled and open circles, respectively. Average +/- SD of two independent biological replicates. WT n=17, *NEK1* KO n=20. Statistics are based on two-tailed, unpaired Student's t-test.

k) Representative images of U-ExM showing the centrosomes of ARPE-19 WT and *NEK1* KO cells stained for α-tubulin and Cep44. Scale bar, 250 nm.

l) Quantification of the length of Cep44 signal at the centrioles from **(k)**. The signal length was normalized to the length of the respective core centriole. For *NEK1* KO cells, only hyperelongated centrioles were quantified. Average +/- SD of two independent biological replicates. WT n=18, *NEK1* KO n=16. Statistics are based on two-tailed, unpaired Student's t-test.

m) Representative images of U-ExM showing the centrosomes of ARPE-19 WT and *NEK1* KO cells stained for α-tubulin and Cep350. Scale bar, 250 nm.

n) Quantification of the length of Cep350 signal at the centrioles from **(m)**. The signal length was normalized to the length of the respective core centriole. For *NEK1* KO cells, only hyperelongated centrioles were quantified. The lower and the upper signal boundaries are indicated by filled and open circles, respectively. Average +/- SD of two independent biological replicates. WT n=30, *NEK1* KO n=26. Statistics are based on two-tailed, unpaired Student's t-test.

o) Representative images of U-ExM showing the centrosomes of ARPE-19 WT and *NEK1* KO cells stained for acetylated tubulin (Ac tub, centriole marker) and C2CD3. Scale bar, 250 nm.

p) Quantification of the length of C2CD3 signal at the centrioles from **(o)**. The signal length was normalized to the length of the respective core centriole. For *NEK1* KO cells, only hyperelongated centrioles were quantified. The lower and the upper signal boundaries are indicated by filled and open circles, respectively. Average +/- SD of two independent biological replicates. WT n=20, *NEK1* KO n=18. Statistics are based on two-tailed, unpaired Student's t-test. Source data are provided as a Source Data file.

Supplementary Figure 4

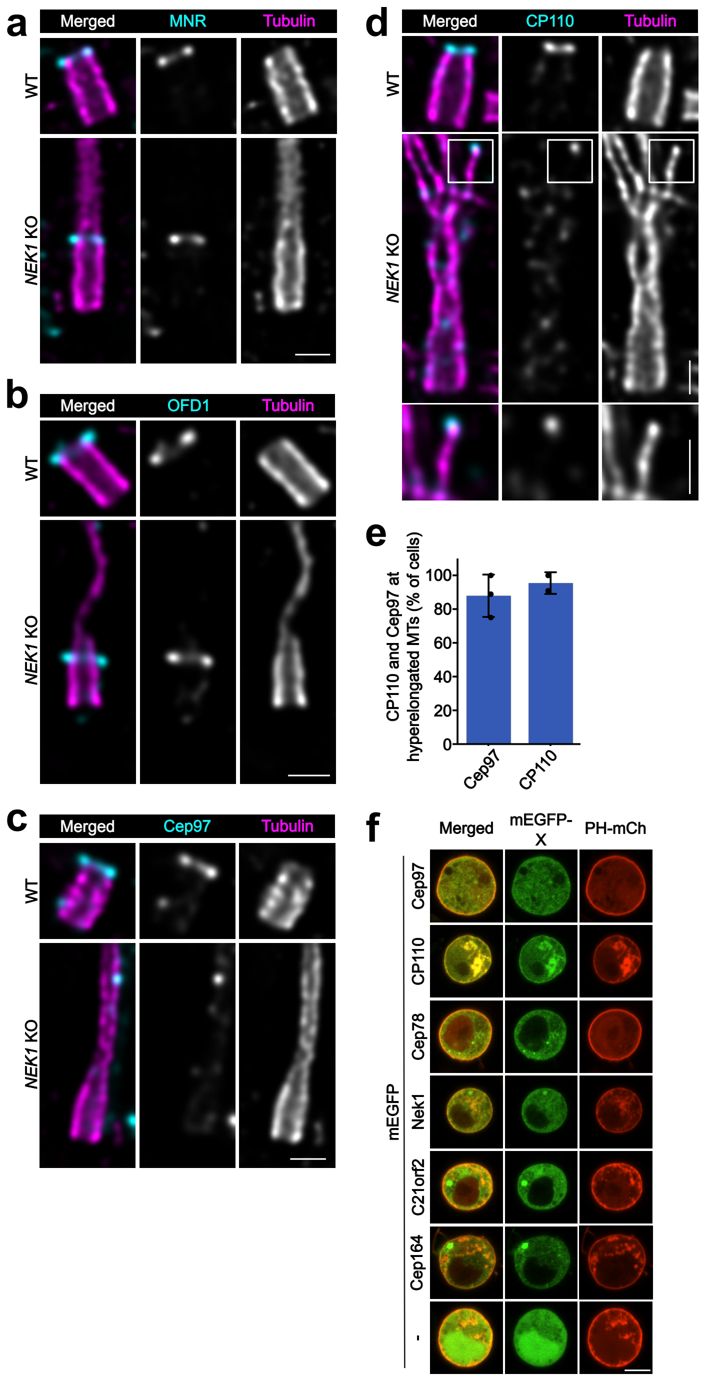

Supplementary Figure 4. Localization of centriolar distal tip proteins in ARPE-19 *NEK1* KO cells.

a-d) U-ExM images show protein localization at representative centrioles of ARPE-19 WT and *NEK1* KO cells from two (**(a)** and **(d)**) or three (**(b)** and **(c)**) independent biological replicates. Scale bar, 250 nm.

e) Quantification of the percentage of *NEK1* KO cells from **(c)** and **(d)** with Cep97 or CP110 at the tips of hyperelongated centriolar MTs. Average +/- SD of three (Cep97) or two (CP110) independent biological replicates. Cep97 n=29, CP110 n=19.

f) Control for the ReLo assay (shown in Fig. 4e). Images of S2R+ cells co-expressing the indicated mEGFP-fusion constructs and PH-mCherry (mCh) as a bait. Scale bar, 5 μm. Representative images from two independent biological replicates. Source data are provided as a Source Data file.

Supplementary Figure 5

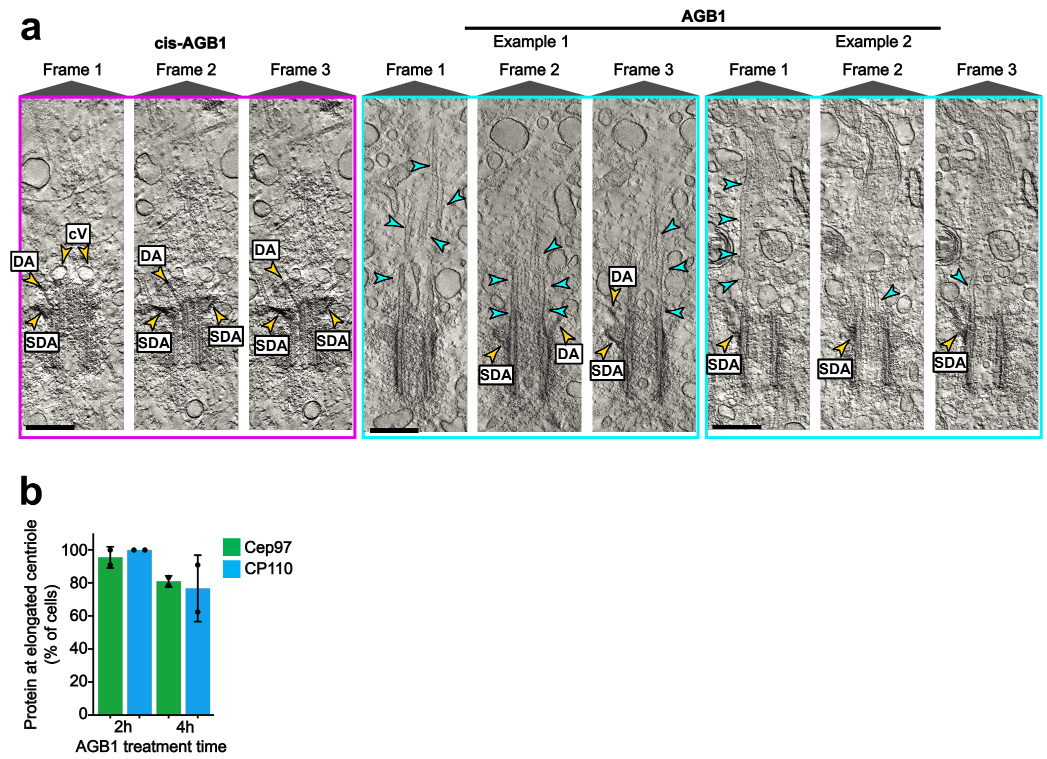

Supplementary Figure 5. Characterization of centrioles in RPE1 *BromoTag-NEK1*.

a) Electron tomograms of RPE1 *BromoTag-NEK1* cells with (cis-AG1, magenta) or without Nek1 (AGB1, cyan) degradation. Cyan arrowheads indicate hyperelongated MTs, yellow arrowheads point to distal appendages (DA), subdistal appendages (SDA), or ciliary vesicles (cV) as indicated. Scale bar, 200 nm. Representative tomograms from 7 (cis-AGB1) or 5 (AGB1) cells.

b) Quantification of the presence of Cep97 and CP110 along the centriolar hyperelongations after 2h and 4h of Nek1 degradation by AGB1 treatment in RPE1 *BromoTag-NEK1* cells. Only centrioles with hyperelongated MTs were considered. Graph shows the average +/- SD from two independent biological replicates. Cep97 2h n=22, Cep97 4h n=26, CP110 2h n=22, CP110 4h n=19. Statistics are based on paired, one-tailed Student's t-test. Source data are provided as a Source Data file.

Supplementary Figure 6

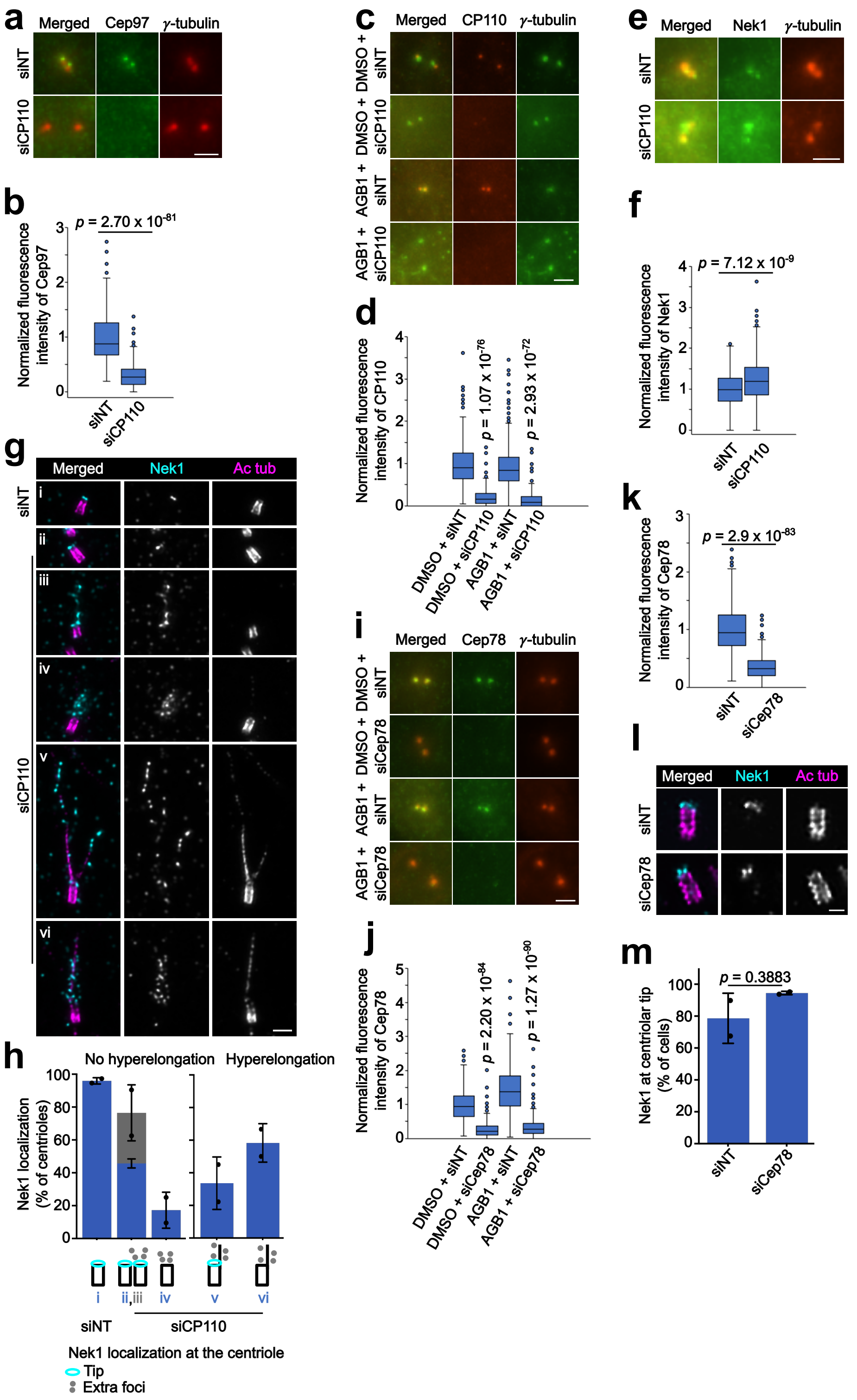

Supplementary Figure 6. Interdependence of protein localization at the centriolar distal tip. **a)** Representative immunofluorescence images of Cep97 at centrosomes (γ-tubulin) in RPE1 *BromoTag-NEK1* cells treated with non-targeting (siNT) or CP110 siRNA. Scale bar, 2 μm.

b) Quantification of **(a)**. siNT n=300, siCP110 n=268. The boxplot shows the median and upper and lower quartiles of three independent biological replicates. Statistics are based on two-tailed, unpaired Student's t-test. Maximum, upper whisker, median, lower whisker, minimum values are as follows: siNT: 2.74, 2.20, 0.88, 0.19, 0.19; siCP110: 1.37, 0.90, 0.27, 0.00, 0.00.

c) Representative immunofluorescence images of centrosomes stained with γ-tubulin and CP110 in RPE1 *BromoTag-NEK1* cells. Cells were treated with solvent control (DMSO, no Nek1 degradation) or AGB1 (Nek1 degradation) in the presence of non-targeting (siNT) or CP110 siRNAs as indicated. Scale bar, 2 μm.

d) Quantification of **(c)**. DMSO+siNT, n=300, DMSO+siCP110 n=282, AGB1+siNT n=300, AGB1+siCP110 n=296. The boxplot shows the median and upper and lower quartiles of three independent biological replicates. Statistics are based on two-tailed, unpaired Student's t-test and refer to the respective DMSO control. Maximum, upper whisker, median, lower whisker, minimum values are as follows: DMSO+siNT: 3.61, 2.43, 0.90, 0.05, 0.05; DMSO+siCP110: 1.38, 0.83, 0.15, 0.00, 0.00; AGB1+siNT: 3.46, 2.12, 0.84, 0.00, 0.00; AGB1+siCP110: 1.35, 0.86, 0.08, 0.00, 0.00.

e) Representative immunofluorescence images of Nek1 at the centrosome (γ-tubulin) in RPE1 *BromoTag-NEK1* cells treated with non-targeting (siNT) or CP110 siRNA. Scale bar, 2 μm.

f) Quantification of **(e)**. siNT n=300, siCP110 n=287. The boxplot shows the median and upper and lower quartiles of three independent biological replicates. Statistics are based on two-tailed, unpaired Student's t-test. Maximum, upper whisker, median, lower whisker, minimum values are as follows: siNT: 2.15, 2.11, 0.98, 0.00, 0.00; siCP110: 3.66, 2.67, 1.19, 0.00, 0.00.

g) Representative U-ExM images of Nek1 at centrioles (acetylated tubulin, Ac tub) in RPE1 *BromoTag-NEK1* cells treated with non-targeting (siNT) or CP110 siRNAs as indicated. The Roman numbers within the panels indicate the different localization patterns as quantified in **(h)**. Scale bar, 500 nm.

h) Quantification of Nek1 localization at centrioles from **(g)** from two independent biological replicates. Cartoons indicate Nek1 localization at the tip (cyan circle) and/or around hyperelongated MTs of centrioles (grey dots) in absence (i-iv) or presence (v, vi) of hyperelongated centriolar MTs, as indicated. Roman numbers refer to the phenotype displayed in **(g)**. siNT n=74, siCP110 no hyperelongation n=37, siCP110 hyperelongation n=38 centrioles. Basal bodies were not considered for quantification. Single data points refer to the total percentage of centrioles within one stacked bar.

i) Representative immunofluorescence images of centrosomes stained with γ-tubulin and Cep78 in RPE1 *BromoTag-NEK1* cells. Cells were treated with solvent control (DMSO, no Nek1 degradation) or AGB1 (Nek1 degradation) in the presence of non-targeting (siNT) or Cep78 siRNAs as indicated. Scale bar, 2 μm.

j) Quantification of **(i)**. DMSO+siNT n=305, DMSO+siCep78 n=302, AGB1+siNT n=300, AGB1+siCep78 n=300. The boxplot shows the median and upper and lower quartiles of three independent biological replicates. Statistics are based on two-tailed, unpaired Student's t-test and

refer to the respective DMSO control. Maximum, upper whisker, median, lower whisker, minimum values are as follows: DMSO+siNT: 2.58, 2.30, 0.94, 0.07, 0.07; DMSO+siCep78: 2.01, 0.98, 0.21, 0.00, 0.00; AGB1+siNT: 4.62, 3.46, 1.37, 0.05, 0.05; AGB1+siCep78: 2.63, 1.19, 0.27, 0.00, 0.00.

k) Immunofluorescence analysis of Cep78 in RPE1 WT cells treated with non-targeting (siNT) or Cep78 siRNA. The boxplot shows the quantification of the fluorescence intensity of Cep78 at centrosomes. The median and upper and lower quartiles of three independent biological replicates are shown. siNT n=300, siCep78 n=287. Statistics are based on two-tailed, unpaired Student's t-test. Maximum, upper whisker, median, lower whisker, minimum values are as follows: siNT: 2.42, 2.11, 0.94, 0.10, 0.10; siCep78: 1.25, 0.87, 0.32, 0.00, 0.00.

l) Representative U-ExM images of Nek1 at centrioles (acetylated tubulin, Ac tub) of RPE1 WT cells depleted of Cep78 of the experiment shown in **(k)**. Scale bar, 250 nm.

m) Quantification of Nek1 localization at the expanded centrioles from **(l)**. The average +/- SD of two independent biological replicates are shown. Basal bodies were not considered for quantification. siNT n=79, siCep78 n=90. Statistics are based on two-tailed, unpaired Student's t-test. Source data are provided as a Source Data file.

Supplementary tables

Supplementary table 1: siRNAs used in this study

<table>
<tr><th>Target</th><th>Sequences (5’ to 3’)</th><th>Manufacturer</th><th>Reference</th></tr>
<tr><td>Cep123</td><td>CCCUGGUUGUUGGAUAUAA</td><td>Ambion Silencer Select</td><td>PMID: 23789104</td></tr>
<tr><td>Cep78</td><td>GAGGAGUUGUCCAGAAAUAUU</td><td>Dharmacon ON-Target plus Standard (A4)</td><td>PMID: 28242748</td></tr>
<tr><td rowspan="4">Cep78_1</td><td>1. GCUGAGAGUCUUCGUAUA</td><td rowspan="4">Dharmacon ON-Target plus Smart pool L-026005-00-0005</td><td rowspan="4"></td></tr>
<tr><td>2. GCGAUAAGAUACAAAGAUG</td></tr>
<tr><td>3. UAGCAAAGGGAUUGAAUAA</td></tr>
<tr><td>4. GAGAGAGGGAUUUAACUAU</td></tr>
<tr><td>CP110</td><td>AAGCAGCAUGAGUAUGCCAGU</td><td>Ambion Silencer Select</td><td>PMID: 40892854</td></tr>
<tr><td rowspan="4">Non-targeting pool (siControl)</td><td>1. UGGUUUACAUGUCGACUAA</td><td rowspan="4">Dharmacon ON-Target plus Non-targeting pool D-001810-10-05</td><td rowspan="4"></td></tr>
<tr><td>2. UGGUUUACAUGUUGUGUGA</td></tr>
<tr><td>3. UGGUUUACAUGUUUUCUGA</td></tr>
<tr><td>4. UGGUUUACAUGUUUUCCUA</td></tr>
</table>

Supplementary table 2: Primary antibodies used in this study

Antibody	Manufacturer	Dilution
Guinea pig anti-Cep123-N	Homemade (PMID: 30131441)	IF: 1:1000
Guinea pig anti-ODF2	Homemade (PMID: 23400999)	U-ExM: 1:800
Mouse anti-acetylated tubulin (clone C3B9)	Homemade (PMID: 2606940)	U-ExM: 1:100
Mouse anti-actin	Chemicon/Millipore #MAB1501	WB: 1:5000
Mouse anti-Centrin	Millipore #04-1624	IF/U-ExM: 1:1000
Mouse anti-Cep350	CL3423; Abcam	U-ExM: 1:500
Mouse anti-CP110	Millipore #MABT1354	IF/U-ExM: 1:1000
Mouse anti-GFP	Roche #11814460001	IF: 1:1000, WB: 1:10000
Mouse anti-GTU88	Sigma Aldrich #T6557	IF: 1:1000
Mouse anti-Sas6	SCBT #sc-81431	IF/U-ExM: 1:50
Rabbit anti-α-tubulin	Proteintech #660311-1-Ig	IF/U-ExM: 1:1000
Rabbit anti-γ-tubulin	Sigma #T5192	IF: 1:1000
Rabbit anti-α-tubulin	Proteintech #11224-1-AP	IF/U-ExM: 1:1000
Rabbit anti-Arl13b	Proteintech #30332-1-AP	IF: 1:1000
Rabbit anti-C2CD3	Sigma-Aldrich #HPA038552	U-ExM: 1:500
Rabbit anti-Centrin	Abcam #ab101332	IF/U-ExM: 1:500
Rabbit anti-Cep120	Proteintech #24067-1-AP	U-ExM: 1:500
Rabbit anti-Cep135	Homemade (PMID: 21059844)	U-ExM: 1:100
Rabbit anti-Cep164	Homemade (PMID: 23253480)	U-ExM: 1:1000
Rabbit anti-Cep44	Homemade (PMID: 32060285)	U-ExM: 1:100
Rabbit anti-Cep78	Bethyl #A301-800A-T	IF/U-ExM: 1:250 to 1:500
Rabbit anti-Cep83	Sigma #HPA038161	U-ExM: 1:1000
Rabbit anti-Cep97	Biomol #A301-947A	IF/U-ExM: 1:300 to 1:600
Rabbit anti-CPAP	Proteintech #11517-1-AP	IF/U-ExM: 1:500
Rabbit anti-Flag	Proteintech #20543-1-AP	WB: 1:3000
Rabbit anti-MNR	Novus Biologicals #NBP1-90929	U-ExM: 1:500
Rabbit anti-Nek1	Abcam # ab229489	U-ExM: 1:500
Rabbit anti-OFD1	Kind gift from Andrew Fry	U-ExM: 1:500
Rabbit anti-POC5	Bethyl #A303-341A-T	U-ExM: 1:500
Sheep anti-Nek1	Homemade (PMID: 37188479)	IF/U-ExM: 1:50 to 1:100
		WB: 1:2000

Supplementary table 3: Secondary antibodies used in this study

**Antibody**	**Manufacturer**	**Dilution**
Donkey anti-mouse AlexaFluor 488	Invitrogen Molecular Probes #A-21202	IF/U-ExM: 1:500
Donkey anti-mouse AlexaFluor 594	Invitrogen Molecular Probes #A-21203	IF/U-ExM: 1:500
Donkey anti-mouse AlexaFluor 647	Invitrogen Molecular Probes #A-31571	IF/U-ExM: 1:500
Donkey anti-rabbit AlexaFluor 488	Invitrogen Molecular Probes #A-21206	IF/U-ExM: 1:500
Donkey anti-rabbit AlexaFluor 594	Invitrogen Molecular Probes #A-21207	IF/U-ExM: 1:500
Donkey anti-rabbit AlexaFluor 647	Invitrogen Molecular Probes #A-31573	IF/U-ExM: 1:500
Donkey anti-sheep AlexaFluor 488	Invitrogen Molecular Probes #A11015	IF/U-ExM: 1:500
Donkey anti-sheep AlexaFluor 546	Invitrogen Molecular Probes #A-21098	IF: 1:500
Goat Abberior STAR 635P anti-mouse	Abberior #ST635P-1001-500UG	IF/U-ExM: 1:500
Goat Abberior STAR 635P anti-rabbit	Abberior #ST635P-1002-500UG	IF/U-ExM: 1:500
Goat anti-guinea pig AlexaFluor 488	Invitrogen Molecular Probes #A-11073	IF: 1:500
Goat anti-guinea pig AlexaFluor Plus 647	Thermo Fisher Scientific # A-21450	IF: 1:500
Goat anti-mouse AlexaFluor 488	Invitrogen Molecular Probes #A11029	IF: 1:500
Goat anti-mouse AlexaFluor 594	Invitrogen Molecular Probes #A11032	IF/U-ExM: 1:500
Goat anti-mouse HRP	Dianova #115-035-003	WB: 1:10000
Goat anti-rabbit AlexaFluor 488	Invitrogen Molecular Probes #A11008	IF/U-ExM: 1:500
Goat anti-rabbit AlexaFluor 594	Invitrogen Molecular Probes #A-11012	IF/U-ExM: 1:500
Goat anti-rabbit HRP	Dianova #111-035-003	WB: 1:10000
Rabbit anti-sheep HRP	Thermo Fisher Scientific #61-8620	WB: 1:10000

Supplementary table 4: Plasmids used in this study

Plasmid	Description	Reference
DU57081	pBABED PURO U6 vector containing a sense guide RNA targeting NEK1 exon 3, along with a puromycin resistance cassette for selection. This construct is intended to be used in conjunction with plasmid 57089. The vector was generated by cloning the sense guide RNA sequence into the BbsI site of plasmid DU48788.	This study
DU57089	pX335 vector containing an antisense guide RNA targeting NEK1 exon 3, along with expression of the CRISPR nickase SpCas9n (D10A). This construct is intended to be used in conjunction with plasmid 57081. The parent vector (Addgene, 42335) was digested with BbsI for guide RNA sequence cloning.	This study
DU74281	pMK RK (kanamycin) vector containing the NEK1 N-terminal GFP–IRES2–BromoTag donor sequence. This construct is intended to be used in conjunction with guide pairs 57081/57089. The pMK RQ (GeneArt) parent plasmid was digested with SalI and EcoRI for cloning.	This study
pAC5.1-eGFP-C21orf2	C21orf2 was subcloned into pAC5-1 mEGFP-CAF1 by HiFi assembly.	This study
pAC5.1-eGFP-CEP164	Cep164 was subcloned into pAC5-1 mEGFP-CAF1 by HiFi assembly.	This study
pAC5.1-eGFP-CEP78	Cep78 was subcloned into pAC5-1 mEGFP-CAF1 by HiFi assembly.	This study
pAC5.1-eGFP-CEP97	Cep97 was subcloned into pAC5-1 mEGFP-CAF1 by HiFi assembly.	This study
pAC5.1-eGFP-CP110	CP110 was subcloned into pAC5-1 mEGFP-CAF1 by HiFi assembly.	This study
pAC5.1-mCherry-PH-NEK1	Nek1 was subcloned into pAC5-1 mCherry-PH-CAF1 by HiFi assembly.	This study
pcDNA5C FRT TO 3xFLAG Nek1	pcDNA5C FRT containing 3xFlag-Nek1. Kind gift from John Rouse (University of Dundee, Scotland).	PMID: 37188479
pESA-SBDonor-TRE3GV-Zeocin	Sleeping Beauty donor plasmid containing TRE3GV promoter and Bleomycin resistance.	PMID: 36315013
pJS038	pESA-SBDonor-TRE3GV-Zeocin containing Cep78. Cep78 was subcloned from pAC5.1-eGFP-CEP78 into pESA-SBDonor-TRE3GV-Zeocin. The vector was cut with Cla1 and EcoR1-HF. The fragments were assembled by HiFi assembly.	This study
pJS039	pESA-SBDonor-TRE3GV-Zeocin containing Cep97. Cep97 was subcloned from pAC5.1-eGFP-CEP97 into pESA-SBDonor-TRE3GV-Zeocin. The vector was cut with Cla1 and EcoR1-HF. The fragments were assembled by HiFi assembly.	This study
pRetro-2xFL-mEGFP-CP110	pRetro-Tet3G containing 2xFlag-mEGFP-CP110. mEGFP-CP110 was subcloned into pRetro-Tet3G containing 2xFlag.	This study

Original uncropped version of the Western Blot shown in Supplementary Figure 2b

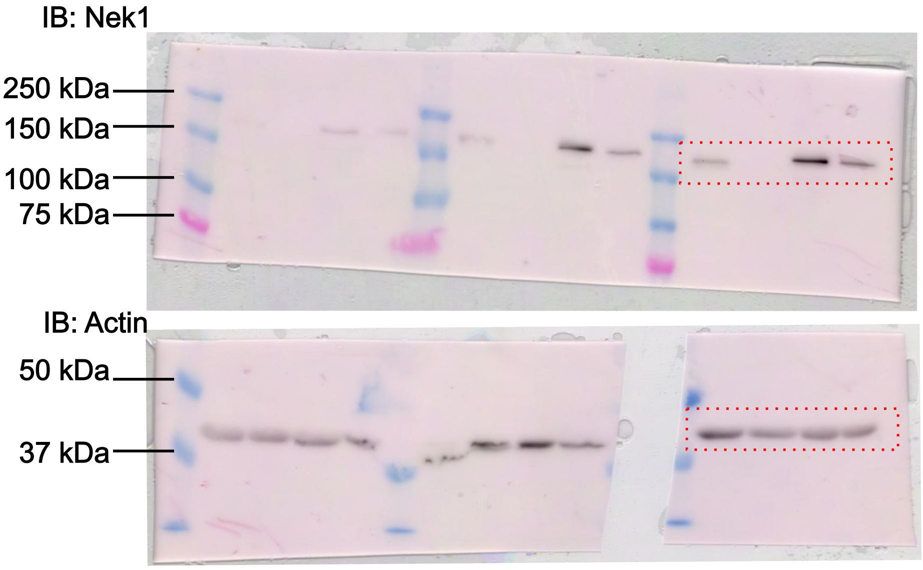

Supplement: Supplementary file 1 — Supplementary Information [file 41467_2026_73560_MOESM1_ESM.pdf]
