# Supplementary material for: Nek1 defines a branch of centriolar microtubule length control parallel to CP110-Cep97
Source: Nat Commun. 2026 Jun 9;17:7330. doi: 10.1038/s41467-026-73560-9 (PMC13402741; doi:10.1038/s41467-026-73560-9)
Supplement: Supplementary file 2 — Reporting Summary [file 41467_2026_73560_MOESM2_ESM.pdf]

nature portfolio

Corresponding author(s): Gislene Pereira, John Rouse

Last updated by author(s): May 4, 2026

Reporting Summary

Nature Portfolio wishes to improve the reproducibility of the work that we publish. This form provides structure for consistency and transparency in reporting. For further information on Nature Portfolio policies, see our Editorial Policies and the Editorial Policy Checklist.

Statistics

For all statistical analyses, confirm that the following items are present in the figure legend, table legend, main text, or Methods section.

n/a	Confirmed	
☐	☒	The exact sample size (*n*) for each experimental group/condition, given as a discrete number and unit of measurement
☐	☒	A statement on whether measurements were taken from distinct samples or whether the same sample was measured repeatedly
☐	☒	The statistical test(s) used AND whether they are one- or two-sided<br>*Only common tests should be described solely by name; describe more complex techniques in the Methods section.*
☒	☐	A description of all covariates tested
☒	☐	A description of any assumptions or corrections, such as tests of normality and adjustment for multiple comparisons
☐	☒	A full description of the statistical parameters including central tendency (e.g. means) or other basic estimates (e.g. regression coefficient) AND variation (e.g. standard deviation) or associated estimates of uncertainty (e.g. confidence intervals)
☐	☒	For null hypothesis testing, the test statistic (e.g. *F*, *t*, *r*) with confidence intervals, effect sizes, degrees of freedom and *P* value noted<br>*Give P values as exact values whenever suitable.*
☒	☐	For Bayesian analysis, information on the choice of priors and Markov chain Monte Carlo settings
☒	☐	For hierarchical and complex designs, identification of the appropriate level for tests and full reporting of outcomes
☒	☐	Estimates of effect sizes (e.g. Cohen's *d*, Pearson's *r*), indicating how they were calculated

Our web collection on statistics for biologists contains articles on many of the points above.

Software and code

Policy information about availability of computer code

Data collection	All immunofluorescence data except for Supplementary Fig. 1e was acquired with Nikon NIS-Elements AR (v. 5.42.06) software and Supplementary Fig. 1e was taken with softWoRxsoftWoRxv6.1.1 Release 5 (AppliedPrecision,GE). U-ExM images were taken either with Leica Falcon LASX Flim v. 3.5.7 (Leica Application Suite X) or Nikon NIS-Elements AR software (v. 5.42.06) as specified in the Methods section. U-ExM images from Leica Falcon microscope were processed with Huygens' Deconvolution software v. 18.10.0p7 (SVIInc.). U-ExM images from Nikon microscope were processed with NIS-Elements AR Analysis 5.30.06. Immunoblots were acquired with Cytiva Amersham ImageQuant800 (v. 2.0.0) For EM images SerialEM(v. 4.1), eTomo (v. 23.0.1/0), IMOD (v. 4.0.29) were used.
Data analysis	For data analysis, Excel version 16.108.1, GraphPad Prism 11.0.0 (93), ImageJ2 version 2.16.0/1.54p were used. Data representation was done by Adobe Illustrator 3.30 and Inkscape Version 1.4.

For manuscripts utilizing custom algorithms or software that are central to the research but not yet described in published literature, software must be made available to editors and reviewers. We strongly encourage code deposition in a community repository (e.g. GitHub). See the Nature Portfolio guidelines for submitting code & software for further information.

Data

Policy information about availability of data

All manuscripts must include a data availability statement. This statement should provide the following information, where applicable:

- Accession codes, unique identifiers, or web links for publicly available datasets
- A description of any restrictions on data availability
- For clinical datasets or third party data, please ensure that the statement adheres to our policy

All relevant data are available in the Supplementary files. Source data supporting the figures are provided with the paper.

Research involving human participants, their data, or biological material

Policy information about studies with human participants or human data. See also policy information about sex, gender (identity/presentation), and sexual orientation and race, ethnicity and racism.

Reporting on sex and gender	N/A
Reporting on race, ethnicity, or other socially relevant groupings	N/A
Population characteristics	N/A
Recruitment	N/A
Ethics oversight	N/A

Note that full information on the approval of the study protocol must also be provided in the manuscript.

Field-specific reporting

Please select the one below that is the best fit for your research. If you are not sure, read the appropriate sections before making your selection.

☒ Life sciences ☐ Behavioural & social sciences ☐ Ecological, evolutionary & environmental sciences

For a reference copy of the document with all sections, see nature.com/documents/nr-reporting-summary-flat.pdf

Life sciences study design

All studies must disclose on these points even when the disclosure is negative.

Sample size	For analysis of immunofluorescent signal intensities, presence/absence of proteins at the centrosome or ciliogenesis, more than 250-300 cells were quantified to obtain higher statistical significance. To determine the procentriole formation by immunoflorescence microscopy, more than 100 cells per condition were quantified. For fluorescence microscopy of localization patterns, n total of 20 cells in different cell cycle phases were acquired to avoid any bias. For U-ExM, minimum of 7 cells per condition were quantified. For TEM, a minimum of 5 cells were acquired. The number of the data points (n) was determined depending on the Standard Deviation (Sd). No formal sample size calculations were performed.
Data exclusions	No data were excluded. Outkiers are indicated in the graphs where applicable.
Replication	For immunofluorescence microscopy and U-ExM, two or more independent biological replicates were performed if replication was required. Western Blot and ReLo experiments were done at least twice independently, Co-IPs were done in biological triplicates.
Randomization	For any microscopy analysis, cells or fields of view for acquisition were chosen randomly to avoid any bias.
Blinding	For immunofluorescence experiments, blinding was not required due to the large sample size. In any other experiments, blinding was not considered necessary due to the use of standardized protocols and quantitative readouts.

Reporting for specific materials, systems and methods

We require information from authors about some types of materials, experimental systems and methods used in many studies. Here, indicate whether each material, system or method listed is relevant to your study. If you are not sure if a list item applies to your research, read the appropriate section before selecting a response.

Materials & experimental systems

n/a	Involved in the study
☐	☒ Antibodies
☐	☒ Eukaryotic cell lines
☒	☐ Palaeontology and archaeology
☒	☐ Animals and other organisms
☒	☐ Clinical data
☒	☐ Dual use research of concern
☒	☐ Plants

Methods

n/a	Involved in the study
☒	☐ ChIP-seq
☒	☐ Flow cytometry
☒	☐ MRI-based neuroimaging

Antibodies

Antibodies used

Guinea pig anti-Cep123-N Homemade (PMID: 30131441) IF: 1:1000
Guinea pig anti-ODF2 Homemade (PMID: 23400999) U-ExM: 1:800
Mouse anti-acetylated tubulin (clone C3B9) Homemade (PMID: 2606940) U-ExM: 1:100
Mouse anti-actin Chemicon/Millipore #MAB1501 WB: 1:5000
Mouse anti-Centrin Millipore #04-1624 IF/U-ExM: 1:1000
Mouse anti-Cep350 CL3423; Abcam U-ExM: 1:500
Mouse anti-CP110 Millipore #MABT1354 IF/U-ExM: 1:1000
Mouse anti-GFP Roche #11814460001 IF: 1:1000, WB: 1:10000
Mouse anti-GTU88 Sigma Aldrich #T6557 IF: 1:1000
Mouse anti-Sas6 SCBT #sc-81431 IF/U-ExM: 1:50
Rabbit anti-a-tubulin Proteintech #660311-1-Ig IF/U-ExM: 1:1000
Rabbit anti-g-tubulin Sigma #T5192 IF: 1:1000
Rabbit anti-a-tubulin Proteintech #11224-1-AP IF/U-ExM: 1:1000
Rabbit anti-Arl13b Proteintech #30332-1-AP IF: 1:1000
Rabbit anti-C2CD3 Sigma-Aldrich #HPA038552 U-ExM: 1:500
Rabbit anti-Centrin Abcam #ab101332 IF/U-ExM: 1:500
Rabbit anti-Cep120 Proteintech #24067-1-AP U-ExM: 1:500
Rabbit anti-Cep135 Homemade (PMID: 21059844) U-ExM: 1:100
Rabbit anti-Cep164 Homemade (PMID: 23253480) U-ExM: 1:1000
Rabbit anti-Cep44 Homemade (PMID: 32060285) U-ExM: 1:100
Rabbit anti-Cep78 Bethyl #A301-800A-T IF/U-ExM: 1:250 to 1:500
Rabbit anti-Cep83 Sigma #HPA038161 U-ExM: 1:1000
Rabbit anti-Cep97 Biomol #A301-947A IF/U-ExM: 1:300 to 1:600
Rabbit anti-CPAP Proteintech #11517-1-AP IF/U-ExM: 1:500
Rabbit anti-Flag Proteintech #20543-1-AP WB: 1:3000
Rabbit anti-MNR Novus Biologicals #NBP1-90929 U-ExM: 1:500
Rabbit anti-Nek1 Abcam # ab229489 U-ExM: 1:500
Rabbit anti-OFD1 (Kind gift from Andrew Fry) U-ExM: 1:500
Rabbit anti-POC5 Bethyl #A303-341A-T U-ExM: 1:500
Sheep anti-Nek1 Homemade (PMID: 37188479) IF/U-ExM: 1:50 to 1:100, WB: 1:2000
Donkey anti-mouse AlexaFluor 488 Invitrogen Molecular Probes #A-21202 IF/U-ExM: 1:500
Donkey anti-mouse AlexaFluor 594 Invitrogen Molecular Probes #A-21203 IF/U-ExM: 1:500
Donkey anti-mouse AlexaFluor 647 Invitrogen Molecular Probes #A-31571 IF/U-ExM: 1:500
Donkey anti-rabbit AlexaFluor 488 Invitrogen Molecular Probes #A-21206 IF/U-ExM: 1:500
Donkey anti-rabbit AlexaFluor 594 Invitrogen Molecular Probes #A-21207 IF/U-ExM: 1:500
Donkey anti-rabbit AlexaFluor 647 Invitrogen Molecular Probes #A-31573 IF/U-ExM: 1:500
Donkey anti-sheep AlexaFluor 488 Invitrogen Molecular Probes #A11015 IF/U-ExM: 1:500
Donkey anti-sheep AlexaFluor 546 Invitrogen Molecular Probes #A-21098 IF: 1:500
Goat Abberior STAR 635P anti-mouse Abberior #ST635P-1001-500UG IF/U-ExM: 1:500
Goat Abberior STAR 635P anti-rabbit Abberior #ST635P-1002-500UG IF/U-ExM: 1:500
Goat anti-guinea pig AlexaFluor 488 Invitrogen Molecular Probes #A-11073 IF: 1:500
Goat anti-guinea pig AlexaFluor Plus 647 Thermo Fisher Scientific # A-21450 IF: 1:500
Goat anti-mouse AlexaFluor 488 Invitrogen Molecular Probes #A11029 IF: 1:500
Goat anti-mouse AlexaFluor 594 Invitrogen Molecular Probes #A11032 IF/U-ExM: 1:500
Goat anti-mouse HRP Dianova #115-035-003 WB: 1:10000
Goat anti-rabbit AlexaFluor 488 Invitrogen Molecular Probes #A11008 IF/U-ExM: 1:500
Goat anti-rabbit AlexaFluor 594 Invitrogen Molecular Probes #A-11012 IF/U-ExM: 1:500
Goat anti-rabbit HRP Dianova #111-035-003 WB: 1:10000
Rabbit anti-sheep HRP Thermo Fisher Scientific #61-8620 WB: 1:10000

Validation

Guinea pig anti-Cep123-N PMID: 30131441
Guinea pig anti-ODF2 PMID: 23400999

Mouse anti-acetylated tubulin (clone C3B9) PMID: 2606940
Mouse anti-actin https://www.merckmillipore.com/DE/de/product/Anti-Actin-Antibody-clone-C4,MM_NF-MAB1501
Mouse anti-Centrin https://www.merckmillipore.com/DE/de/product/Anti-Centrin-Antibody-clone-20H5,MM_NF-04-1624?ReferrerURL=https%3A%2F%2Fwww.google.com%2F
Mouse anti-Cep350 https://www.abcam.com/en-us/products/primary-antibodies/cep350-antibody-cl3423-ab219831
Mouse anti-CP110 https://www.merckmillipore.com/DE/de/product/Anti-CP110-Antibody-clone-140-195-5,MM_NF-MABT1354
Mouse anti-GFP https://www.sigmaaldrich.com/DE/de/product/roche/11814460001?srsltid=AfmBOori4hovGtseFU6MF4FPPmjY1XRT4aLtc94tkvGpruAiJRrWlc8n
Mouse anti-GTU88 https://www.sigmaaldrich.com/DE/de/product/sigma/t6557?utm_source=google&utm_medium=cpc&utm_id=21480163361&utm_campaign=%7Bcampaignname%7D&utm_content=165772949198&utm_term=t6557%20sigma&gad_source=1&gad_campaignid=21480163361&gbraid=0AAAAAD8kLQRVBTEsGrNXDI6WX3WkgsuUQ&gclid=Cj0KCQjw2MbPBhCSARIsAP3jP9yuTENmTUT1tqdbt0cmmE8g8f9RLfmjkc0r5Fh1AQomSSwNsqBGyugaAvndEALw_wcB
Mouse anti-Sas6 https://www.scbt.com/p/sas-6-antibody-91-390-21?srsltid=AfmBOooiPCUB4_4iBCu2atDSZY2B5N_BHfK0YAujShuLnZZVhbswKnBr
Rabbit anti-a-tubulin https://www.ptglab.com/de/products/tubulin-Alpha-Antibody-66031-1-Ig.htm?srsltid=AfmBOorMrYoxVosAZew6MvWyd7vxX4bThY-gpfxDLQtBzVwHbS0kmfMU
Rabbit anti-g-tubulin https://www.sigmaaldrich.com/DE/de/product/sigma/t5192?srsltid=AfmBOoq5tLe6TmbZOSgMrJLY9bIHpeSWfYG2WCek83z0G4jYKKSA8xuK
Rabbit anti-a-tubulin https://www.ptglab.com/de/products/TUBA1B-Antibody-11224-1-AP.htm?srsltid=AfmBOor64dS_A-WhFJHdO7FB1ar2Qz_b3HGqM1bMPjmW-bVM5WZdYsi2
Rabbit anti-Arl13b https://www.ptglab.com/products/ARL13B-Antibody-30332-1-AP.htm?srsltid=AfmBOopucOhCKcMcGqd_2gdHXiySaCOGFSpp6uHMh2PL1R4n1KKiCS5h
Rabbit anti-C2CD3 https://www.sigmaaldrich.com/DE/de/product/sigma/hpa038552?srsltid=AfmBOoqQySqPmCPi_77g2SUMRc-n2L7Isq0msny1GlN5pX3_cPfoVvDg
Rabbit anti-Centrin https://www.abcam.com/en-us/products/primary-antibodies/centrin-1-antibody-ab101332
Rabbit anti-Cep120 https://www.ptglab.com/de/products/CEP120-Antibody-24067-1-AP.htm?srsltid=AfmBOopBI7ZWRUYFJ1Nf4yWERmX8TJVCy8HLY_ia4U9lwWcJFkfeqs4q
Rabbit anti-Cep135 PMID: 21059844
Rabbit anti-Cep164 PMID: 23253480
Rabbit anti-Cep44 PMID: 32060285
Rabbit anti-Cep78 https://www.biomol.com/de/produkte/antikoerper/primaerantikoerper/allgemein/anti-cep78-a301-800a-t
Rabbit anti-Cep83 https://www.sigmaaldrich.com/DE/de/product/sigma/hpa038161?srsltid=AfmBOorOSdQuFJZc10BqfTAGjNn56S93Lv9RnzSsoupHlNiB8IZuXwS_
Rabbit anti-Cep97 https://www.biomol.com/products/antibodies/primary-antibodies/general/anti-cep97-a301-947a-t?number=A301-947A
Rabbit anti-CPAP https://www.ptglab.com/de/products/CENPJ-Antibody-11517-1-AP.htm?srsltid=AfmBOoqbrpJfSPyKwkYi-qZPYxZVBYVODqaqoStKn1fHtOByplet1wTO
Rabbit anti-Flag https://www.ptglab.com/de/products/Flag-Tag-Antibody-20543-1-AP.htm?srsltid=AfmBOopFnJtJqEep76PgBv8al8wyUunzF92Ogce2lZvNHvAx-8FlSv2C
Rabbit anti-MNR https://www.novusbio.com/products/kiaa0753-mnr-antibody_nbp1-90929?srsltid=AfmBOorrYoqDLDfS8UH3_ZM7H3TPcot6R2M0min4iUmGKIO5psWNNL2E
Rabbit anti-Nek1 https://www.abcam.com/en-us/products/primary-antibodies/nek1-antibody-ab229489
Rabbit anti-OFD1 Kind gift from Andrew Fry
Rabbit anti-POC5 https://www.fortislife.com/products/primary-antibodies/rabbit-anti-poc5-antibody/BETHYL-A303-341
Sheep anti-Nek1 PMID: 37188479

Eukaryotic cell lines

Policy information about cell lines and Sex and Gender in Research

Cell line source(s)	hTERT-immortalized RPE1 (ATCC CRL-4000)<br>ARPE-19, ARPE-19 NEK1 KO and NEK1 KO stably expressing either NEK1 WT or NEK1 D146A (Gregorczyk et al., 2023)<br>RPE1 BromoTag-NEK1<br>Hek293T (ATCC CRL-3216) stably expressing Tet3G<br>Drosophila S2R+ (kind gift from Mandy Jeske, University of Heidelberg, Germany)<br>RPE1 TP53 KO (Kind gift from Bryan Tsou, Fong et al. 2016)<br>RPE1 ODF2 KO (Viol et al., 2020)<br>RPE1 CEP83 KO (Laboratory stock)<br>RPE1 TP53 KO CEP350 KO (Karasu et al., 2022)
Authentication	No authentication of cell lines was performed.
Mycoplasma contamination	All cell lines have been regularly checked against Mycoplasma using Eurofins Genomics with negative results.
Commonly misidentified lines (See ICLAC register)	No cell lines listed as commonly misidentified were used in this study.

Plants

Seed stocks	N/A
Novel plant genotypes	N/A
Authentication	N/A
