# Supplementary material for: Nek1 defines a branch of centriolar microtubule length control parallel to CP110-Cep97
Source: Nat Commun. 2026 Jun 9;17:7330. doi: 10.1038/s41467-026-73560-9 (PMC13402741; doi:10.1038/s41467-026-73560-9)
Supplement: Supplementary file 3 — Transparent Peer Review file [file 41467_2026_73560_MOESM3_ESM.pdf]

nature portfolio

Peer Review File

Nek1 Defines a New Branch of Centriolar Microtubule Length Control Parallel to CP110-Cep97

Corresponding Author: Professor Gislene Pereira

This file contains all reviewer reports in order by version, followed by all author rebuttals in order by version.

Version 0:

Reviewer comments:

Reviewer #1

(Remarks to the Author)
In this study, Streubel et al. identify NEK1 as a novel key regulator of centriole length, acting in parallel with the well-characterized CP110–CEP97 distal complex. Previous work has localized NEK1 to the centrosome and basal body, where it contributes to the coordination of ciliogenesis and cell-cycle progression and to the maintenance of centrosome/centriole integrity. Here, by applying Ultrastructure Expansion Microscopy (U-ExM), the authors significantly enhance spatial resolution and precisely define the relative localization of NEK1 at the distal end of centrioles.

The authors demonstrate that loss of NEK1 leads to hyperelongation of centriolar microtubules from the distal tip, without disrupting the CP110–CEP97 distal complex. Importantly, they provide evidence that NEK1 and the CP110–CEP97 complex act synergistically, functioning in parallel pathways to control centriole length and to restrict excessive microtubule extension from both mother and daughter centrioles. Furthermore, the study shows that NEK1 is removed from the basal body during ciliogenesis in a CEP78-dependent manner, a process that closely mirrors the removal of CP110–CEP97, suggesting mechanistic parallels in the regulation of distal centriole remodeling during cilium formation. Although the molecular mechanism remains incompletely resolved, this work will be of significant interest to the centrosome and centriole research community, as it uncovers a previously unrecognized role for NEK1 in centriole length control, acting alongside the established CP110–CEP97 pathway.

For the most part, the experiments are carefully executed and well analyzed. Prior to publication, I recommend that the authors address the following major and minor points to further strengthen the manuscript.

Major:

1. NEK1 localization: Using U-ExM, the authors show that NEK1 localizes to the distal tip of both mother and daughter centrioles, with an additional weaker signal along the centriolar walls, resembling the tubulin labeling pattern. The specificity of the signal appears convincing, as it is lost in NEK1-deficient cells. However, the NEK1 signal is noticeably weaker when co-stained with acetylated tubulin, compared with the staining shown using tubulin labeling. It would be important to visualize NEK1 localization in both WT and NEK1 KO cells using tubulin (rather than acetylated tubulin) as the centriolar marker (Fig.S1a) and to quantify NEK1 localization relative to tubulin. This additional information would strengthen the conclusion regarding NEK1's precise subcentriolar localization and ensure that the observed differences in signal intensity are not due to technical limitations of the antibody combinations used.

2. Radial position of NEK1: In lines 130-133, the authors describe NEK1 forming dot-like structures at the centriolar tip that most frequently arrange into an incomplete, C-shaped ring, with less frequent configurations ranging from isolated dots to an almost complete ring (Fig. 1c). While these observations are intriguing, the radial localization of NEK1 remains difficult to interpret based on the current data. In particular, the top-view images in Fig. 1c appear to have lower spatial resolution compared with those shown in Fig. 1a, which were acquired using a Leica TCS SP8 confocal microscope, whereas Fig. 1c was acquired on a Nikon AX confocal. In addition, it would be important to verify that the dot-like appearance is not due to low antibody affinity. To more clearly define the radial distribution of NEK1 at the distal centriole, it would be helpful to acquire top-view images of NEK1(and test different antibodies) co-stained with tubulin or acetylated tubulin with the same imaging quality that panel 1a. Such imaging would likely improve resolution and facilitate a more accurate assessment of

NEK1’s spatial organization. In addition, these data could allow quantitative measurements of the NEK1 signal diameter, enabling direct comparison with known distal centriole ring-like structures such as CP110, CEP97, or CEP78, and thereby strengthening conclusions regarding NEK1’s structural arrangement at the centriole tip.

3. In line 135, the authors state that NEK1 “partially co-localizes” with the distal appendage protein CEP83. However, based on the staining shown in Fig. 1d and the corresponding intensity profiles in Fig. 1e, NEK1 and CEP83 do not appear to occupy the same spatial position. Instead, NEK1 localizes distally relative to the distal appendage marker CEP83, positioning it above the distal appendage region. Because the term co-localization implies overlapping or coincident localization of two proteins, its use in this context may be misleading. Accordingly, I suggest revising the wording in the main text and figure legend (Fig. 1d) to reflect relative localization rather than co-localization (e.g., “U-ExM showing the relative localization of NEK1 with CEP97 or CEP83”). Based on the presented data, co-localization is supported for NEK1 and CEP97, but not for NEK1 and CEP83.

4. The manuscript states that the DISCO complex localizes at the distal tip of centrioles. However, published work shows that the DISCO complex resides slightly below the distal end, whereas CP110 and CEP97 cap the very distal ends of centriolar microtubules (see: https://doi.org/10.1371/journal.pbio.3001782). Similarly, CEP83 localizes at the same level as the DISCO complex. The current wording is therefore misleading and should be revised. Importantly, the authors’ own results are actually consistent with this organization, as the DISCO complex does not regulate microtubule elongation per se, and its loss primarily affects overall centriole length rather than distal microtubule extension.

5. While the representative images in Fig. 1a, 3a, Fig. 4a-b, Fig 5g, Fig. S3f-g are informative, the analysis would be significantly strengthened by the inclusion of quantitative analyses. Also, for figure 1d-e, only one centriole is analysed. I strongly recommend including graphs quantifying centriole length, protein distribution, and/or the proportion of centrioles showing altered localization of specific proteins in WT versus NEK1-deficient cells. Such quantification would allow a more objective assessment of localisation, phenotype penetrance and robustness.

6. The elongation of centriolar microtubules observed upon NEK1 loss is striking. In the EM images, it appears that primarily the A-microtubule elongates rather than the full triplet. Can the authors directly assess this point using their tomographic datasets? In addition, arrows and color coding in the EM figures are not clearly explained in the legends, making interpretation difficult. The legends should be clarified accordingly.

7. The duplication phenotype should be analyzed more rigorously by quantitatively comparing the number of procentrioles in WT versus NEK1 KO cells. Also, although CPAP localization is examined at procentrioles in NEK1 KO cells, the most prominent elongation phenotype affects the mother centriole. Given CPAP’s central role in centriole elongation and its distal localisation, it would be important to analyze CPAP localization along the over-elongated centriolar microtubules in NEK1-deficient cells.

8- It would be informative to explore by expansion microscopy whether NEK1 remains associated with elongated centriolar microtubules in siCP110, siCEP97, or siCEP78 conditions. Such experiments could further clarify pathway relationships between NEK1 and the CP110–CEP97 axis as illustrated in the final model.

9. If feasible, it would be informative to test the effect of pharmacological inhibition of NEK1 or expression of a kinase-dead NEK1 mutant to directly assess whether NEK1 kinase activity is required for regulating centriolar microtubule elongation.

Minor:

Line 49: Centrioles are connected at their proximal ends, not distal ends.
Line 50: The statement that the two centrioles "differ in composition and function" should be nuanced, as mother and daughter centrioles are largely similar, with defined but limited compositional differences.
Line 59: "G2/S" is unclear. Centriole duplication initiates at the G1/S transition and elongation continues through G2 and mitosis.
Line 109: In addition to the distal tip, NEK1 localization along the centriole wall should be explicitly mentioned.
Line 682: Typographical errors: "6g → 6f; 7d → 7c
Line 944: The scale bar of "1.250 nm" appears to be incorrect.

Reviewer #2

(Remarks to the Author)
This is an extremely nice paper assigning a new function to Nek1 in a previously unanticipated pathway for regulation of the lengths of centriolar associated microtubules. The finding that Nek1 is working alongside the CEP110 complex to regulate the length of centriolar microtubules is a seminal discovery that should be published in Nature Communications.
The findings of the paper are very clearly described and represent the results of a well-thought out series of experiments. I have no changes to suggest to the body of the paper that can be published as is.
I do question the title as I am not sure convinced that the authors are describing centriole length control per se – after all the microtubule length extension occurs at the distal end of what looks like an (almost) normal centriole. In perhaps the same vein, the authors might like to note a series of papers from Benedicte Durand’s lab describing how the absence of the transition zone results in extension of centriolar microtubules in the male germ line. The authors might like to discuss the relevance of such studies to their current findings.

Version 1:

Reviewer comments:

Reviewer #1

(Remarks to the Author)
I appreciate the authors' efforts in revising the manuscript and responding to the earlier comments in detail. The manuscript has improved, and most of the previous concerns have been addressed appropriately.

The authors have done an excellent job revising the manuscript. I recommend the revised manuscript for publication.

Open Access This Peer Review File is licensed under a Creative Commons Attribution 4.0 International License, which permits use, sharing, adaptation, distribution and reproduction in any medium or format, as long as you give appropriate credit to the original author(s) and the source, provide a link to the Creative Commons license, and indicate if changes were made.
In cases where reviewers are anonymous, credit should be given to 'Anonymous Referee' and the source.
The images or other third party material in this Peer Review File are included in the article’s Creative Commons license, unless indicated otherwise in a credit line to the material. If material is not included in the article’s Creative Commons license and your intended use is not permitted by statutory regulation or exceeds the permitted use, you will need to obtain permission directly from the copyright holder.
To view a copy of this license, visit https://creativecommons.org/licenses/by/4.0/

Response letter to reviewers

We would like to thank both reviewers for the constructive comments on our MS.

Reviewer #1 (Remarks to the Author):

In this study, Streubel et al. identify NEK1 as a novel key regulator of centriole length, acting in parallel with the well-characterized CP110–CEP97 distal complex. Previous work has localized NEK1 to the centrosome and basal body, where it contributes to the coordination of ciliogenesis and cell-cycle progression and to the maintenance of centrosome/centriole integrity. Here, by applying Ultrastructure Expansion Microscopy (U-ExM), the authors significantly enhance spatial resolution and precisely define the relative localization of NEK1 at the distal end of centrioles.

The authors demonstrate that loss of NEK1 leads to hyperelongation of centriolar microtubules from the distal tip, without disrupting the CP110–CEP97 distal complex. Importantly, they provide evidence that NEK1 and the CP110–CEP97 complex act synergistically, functioning in parallel pathways to control centriole length and to restrict excessive microtubule extension from both mother and daughter centrioles. Furthermore, the study shows that NEK1 is removed from the basal body during ciliogenesis in a CEP78-dependent manner, a process that closely mirrors the removal of CP110–CEP97, suggesting mechanistic parallels in the regulation of distal centriole remodeling during cilium formation. Although the molecular mechanism remains incompletely resolved, this work will be of significant interest to the centrosome and centriole research community, as it uncovers a previously unrecognized role for NEK1 in centriole length control, acting alongside the established CP110–CEP97 pathway.

For the most part, the experiments are carefully executed and well analyzed. Prior to publication, I recommend that the authors address the following major and minor points to further strengthen the manuscript.

Major:

1. NEK1 localization: Using U-ExM, the authors show that NEK1 localizes to the distal tip of both mother and daughter centrioles, with an additional weaker signal along the centriolar walls, resembling the tubulin labeling pattern. The specificity of the signal appears convincing, as it is lost in NEK1-deficient cells. However, the NEK1 signal is noticeably weaker when co-stained with acetylated tubulin, compared with the staining shown using tubulin labeling. It would be important to visualize NEK1 localization in both WT and NEK1 KO cells using tubulin (rather than acetylated tubulin) as the centriolar marker (Fig.S1a) and

to quantify NEK1 localization relative to tubulin. This additional information would strengthen the conclusion regarding NEK1's precise subcentriolar localization and ensure that the observed differences in signal intensity are not due to technical limitations of the antibody combinations used.

Thank you for pointing out this discrepancy and for the helpful suggestion. In response, we repeated the experiments and performed co-staining of Nek1 with anti-tubulin, and imaged centrioles using a Leica SP8 confocal microscope. In contrast to our original result (Fig. 1a), we could not reproduce detectable centriolar microtubule wall staining for Nek1 when co-stained with anti-tubulin. The main experimental change was the use of a newly purchased batch of the anti-tubulin antibody from the same supplier as before. Using this new batch, we mainly detect Nek1 at the distal end of the centrioles with the sheep anti-Nek1 antibody. This centriolar tip signal was absent in the Nek1 knockout cell line (Supplementary Fig. 1a), supporting its specificity. We suspect that the weak centriolar wall staining previously observed may have resulted from antibody crosstalk, specifically between the earlier anti-tubulin batch and anti-sheep IgGs. As the KO control was performed with anti-acetylated tubulin, this cross-reaction remained unnoticed. Unfortunately, we have run out of the original anti-tubulin antibody, preventing additional validation experiments with that reagent. Given that, under the updated staining conditions, the centriolar wall signal for Nek1 (tested with different centriolar microtubule markers and Nek1 antibodies) was extremely dim and near or below the detection limit on both Leica and Nikon systems, we do not consider centriolar wall localization of Nek1 sufficiently supported. Accordingly, we are not emphasizing this localization in the current version of the manuscript. Importantly, a comparable Nek1 staining pattern at the centriolar distal tip was also obtained with an independent, commercially available anti-Nek1 antibody (rabbit anti-Nek1, abcam), which is now included in Supplementary Fig. 1b.

2. Radial position of NEK1: In lines 130-133, the authors describe NEK1 forming dot-like structures at the centriolar tip that most frequently arrange into an incomplete, C-shaped ring, with less frequent configurations ranging from isolated dots to an almost complete ring (Fig. 1c). While these observations are intriguing, the radial localization of NEK1 remains difficult to interpret based on the current data. In particular, the top-view images in Fig. 1c appear to have lower spatial resolution compared with those shown in Fig. 1a, which were acquired using a Leica TCS SP8 confocal microscope, whereas Fig. 1c was acquired on a Nikon AX confocal. In addition, it would be important to verify that the dot-like appearance is not due to low antibody affinity. To more clearly define the radial distribution of NEK1 at the distal centriole, it would be helpful to acquire top-view images of NEK1(and test different antibodies) co-stained with tubulin or acetylated tubulin with the same imaging quality that panel 1a. Such imaging would likely improve resolution and facilitate a more accurate assessment of NEK1's spatial organization. In addition, these data could allow quantitative measurements of the NEK1 signal diameter, enabling direct comparison with known distal centriole ring-like structures such as CP110, CEP97, or CEP78, and thereby strengthening conclusions regarding NEK1's structural arrangement at the centriole tip.

As suggested, we have now analyzed and quantified the radial distribution of Nek1 relative to tubulin, Cep97, and Cep78. The Nek1 ring diameter was similar to that of tubulin and Cep97, but significantly different from Cep78 (Fig. 1d). We further acquired images of Nek1 at centrioles from the top view on the Leica SP8 microscope for improved image quality. The improved images are shown in Fig. 1c. We also imaged Nek1 using an independent antibody (rabbit anti-Nek1 from Abcam). The same pattern was observed, with a dot-like signal frequently arranged as partial (C-shaped) or complete circles in top views (now shown in Supplementary Fig. 1c). Please note that with this antibody, we could co-stain only with mouse anti–acetylated tubulin, and not with rabbit anti-tubulin, due to species compatibility. Increasing the Nek1 antibody concentration did not change the punctate appearance of the signal, but it increased signal intensity and the proportion of centrioles displaying complete or incomplete circular arrangements (as shown in the new quantifications in Fig. 1c).

3. In line 135, the authors state that NEK1 "partially co-localizes" with the distal appendage protein CEP83. However, based on the staining shown in Fig. 1d and the corresponding intensity profiles in Fig. 1e, NEK1 and CEP83 do not appear to occupy the same spatial position. Instead, NEK1 localizes distally relative to the distal appendage marker CEP83, positioning it above the distal appendage region. Because the term co-localization implies overlapping or coincident localization of two proteins, its use in this context may be misleading. Accordingly, I suggest revising the wording in the main text and figure legend (Fig. 1d) to reflect relative localization rather than co-localization (e.g., "U-ExM showing the relative localization of NEK1 with CEP97 or CEP83"). Based on the presented data, co-localization is supported for NEK1 and CEP97, but not for NEK1 and CEP83.

We have revised the text to reflect that the relative localization of Nek1 is closer to Cep97 than to Cep83 in lateral views (page 6 and legend Figure 1 e-g). We have also replaced the line scan quantifications with bar graphs in Fig. 1e (now Fig. 1f and 1g) to include statistical analysis comparing the relative localization of Nek1-Cep97 and Nek1-Cep83.

4. The manuscript states that the DISCO complex localizes at the distal tip of centrioles. However, published work shows that the DISCO complex resides slightly below the distal end, whereas CP110 and CEP97 cap the very distal ends of centriolar microtubules (see: https://doi.org/10.1371/journal.pbio.3001782). Similarly, CEP83 localizes at the same level as the DISCO complex. The current wording is therefore misleading and should be revised. Importantly, the authors' own results are actually consistent with this organization, as the DISCO complex does not regulate microtubule elongation per se, and its loss primarily affects overall centriole length rather than distal microtubule extension.

We agree with the reviewer and have revised the text accordingly (page 4). We now state that DISCO complex components (Cep90, MNR, and OFD1) localize to the distal tip region slightly below the distal end of centrioles and at the same level of distal appendages,

consistent with published work, whereas CP110-Cep97 cap the very distal ends of the centriolar microtubules.

5. While the representative images in Fig. 1a, 3a, Fig. 4a-b, Fig 5g, Fig. S3f-g are informative, the analysis would be significantly strengthened by the inclusion of quantitative analyses. Also, for figure 1d-e, only one centriole is analysed. I strongly recommend including graphs quantifying centriole length, protein distribution, and/or the proportion of centrioles showing altered localization of specific proteins in WT versus NEK1-deficient cells. Such quantification would allow a more objective assessment of localisation, phenotype penetrance and robustness.

As requested, we have now added quantitative analyses of protein distribution for the data shown in Fig. 1a, Fig. 3a, Fig. 4a,b, Fig. 5g, and Fig. S3f,g. These quantifications are included in the revised manuscript in Fig. 5h and Supplementary Fig. 3d, 3f, 3h, 3j, 3l, 3n, 3p, and 4e. In addition, we now quantify multiple centrioles (rather than a single example) for the analyses previously shown in Fig. 1d,e, and include the corresponding statistics.

6. The elongation of centriolar microtubules observed upon NEK1 loss is striking. In the EM images, it appears that primarily the A-microtubule elongates rather than the full triplet. Can the authors directly assess this point using their tomographic datasets? In addition, arrows and color coding in the EM figures are not clearly explained in the legends, making interpretation difficult. The legends should be clarified accordingly.

Unfortunately, our conventional EM images and the tomographic datasets did not provide sufficient resolution and/or consistent viewing angles to unambiguously distinguish whether the elongating structure corresponds specifically to the A-tubule versus an extending doublet or triplet. In particular, the relevant distal regions were often captured in orientations where individual microtubule subunits could not be reliably segmented across multiple slices, and the contrast at the distal end was not robust enough to support a definitive conclusion. Therefore, we cannot directly assess from our current data whether elongation occurs exclusively via the A-microtubule or involves extension of doublets/triplets.

7. The duplication phenotype should be analyzed more rigorously by quantitatively comparing the number of procentrioles in WT versus NEK1 KO cells. Also, although CPAP localization is examined at procentrioles in NEK1 KO cells, the most prominent elongation phenotype affects the mother centriole. Given CPAP's central role in centriole elongation and its distal localisation, it would be important to analyze CPAP localization along the over-elongated centriolar microtubules in NEK1-deficient cells.

To better assess the centriole duplication phenotype, we compared the number of procentrioles (Sas6) in S phase (EdU) in WT and *NEK1* KO cells by immunofluorescence microscopy. Both WT and *NEK1* KO cells formed procentrioles to a similar extent. The data are now included in Supplementary Fig. 3a and 3b. To confirm this result, we also checked

the number of procentrioles (Sas6 or CPAP) in S phase cells by U-ExM. This showed that the majority of the cells formed one procentriole per centriole. These data are now included in the text (page 9).
In addition, we have now quantified CPAP localization at mother centrioles in WT and *NEK1* KO cells and did not detect a significant difference between the two conditions. In both cases, CPAP localized at the proximal region and at the distal end of fully elongated centrioles. Importantly, in *NEK1* KO cells, CPAP did not extend along the hyperelongated centriolar microtubules but remained at the proximal and distal ends. We have included these quantifications and representative images in the revised manuscript (Supplementary Fig. 3e and 3f).

8- It would be informative to explore by expansion microscopy whether NEK1 remains associated with elongated centriolar microtubules in siCP110, siCEP97, or siCEP78 conditions. Such experiments could further clarify pathway relationships between NEK1 and the CP110–CEP97 axis as illustrated in the final model.

We have addressed this point by analyzing Nek1 localization after depletion of Cep78 and CP110 using expansion microscopy. We did not include siCep97 because, in our hands, Cep97 knockdown was consistently less efficient than CP110 knockdown and therefore did not allow a robust interpretation. Moreover, since Cep97 centriolar levels also decrease substantially upon CP110 depletion (shown in supplemental figure 6a, b), we focused our analysis on siCP110.

In the absence of Cep78, Nek1 remained concentrated at the distal tips of centrioles (data included in Supplementary Fig. 6k to 6m). For siCP110, the outcome was more heterogeneous. In centrioles that did not show microtubule hyperelongation, Nek1 persisted at the distal tip in the majority of centrioles. In addition, in ~40% of these centrioles, Nek1 accumulated as puncta in the vicinity of the centriole. In hyperelongated centrioles, Nek1 was still detected at the distal tip in ~40% of cases; however, in the remaining centrioles, Nek1 was not detected at the distal tip. Additionally, Nek1 foci appeared along or in close proximity to the hyperelongated centriolar MTs in 90% of the cells (data included in Supplementary Fig. 6g and 6h). Together, these data indicate that Nek1 distal tip localization is retained in non-elongated centrioles in the absence of Cep78 or CP110. However, centriolar microtubule hyperelongation (observed upon CP110 depletion) frequently displaces Nek1 from the centriolar tip, redistributing it along or in close proximity to the hyperelongated centriolar microtubules. These results support the conclusion that Nek1 can localize to the distal tip independently of CP110-Cep97 and Cep78, consistent with the pathway relationships proposed in our model (discussed in page 18-19).

9. If feasible, it would be informative to test the effect of pharmacological inhibition of NEK1 or expression of a kinase-dead NEK1 mutant to directly assess whether NEK1 kinase activity is required for regulating centriolar microtubule elongation.

We performed this experiment using *NEK1* KO cells expressing a kinase-dead *NEK1* mutant. Cells expressing kinase-dead *NEK1* displayed a centriolar microtubule hyperelongation phenotype that was less pronounced than in *NEK1* KO cells, yet stronger than in the WT control. This intermediate phenotype suggests that Nek1's kinase activity is required at least in part to suppress centriolar MT hyperelongation. This data is now shown in Supplementary Fig. 2b and 2c.

Minor:

Line 49: Centrioles are connected at their proximal ends, not distal ends.
Corrected.

Line 50: The statement that the two centrioles "differ in composition and function" should be nuanced, as mother and daughter centrioles are largely similar, with defined but limited compositional differences.
Corrected.

Line 59: "G2/S" is unclear. Centriole duplication initiates at the G1/S transition and elongation continues through G2 and mitosis.
Corrected – we meant the G1/S phase transition, when centriole duplication starts.

Line 109: In addition to the distal tip, NEK1 localization along the centriole wall should be explicitly mentioned.
Based on our additional staining controls and antibody validation, we no longer consider Nek1 to localize along the centriolar microtubule wall.

Line 682: Typographical errors: "6g → 6f; 7d → 7c
Corrected

Line 944: The scale bar of "1.250 nm" appears to be incorrect.
We have now replaced this image and corrected the scale bar accordingly.

Reviewer #2 (Remarks to the Author):

This is an extremely nice paper assigning a new function to Nek1 in a previously unanticipated pathway for regulation of the lengths of centriolar associated microtubules. The finding that Nek1 is working alongside the CEP110 complex to regulate the length of

centriolar microtubules is a seminal discovery that should be published in Nature Communications.
The findings of the paper are very clearly described and represent the results of a well-thought out series of experiments. I have no changes to suggest to the body of the paper that can be published as is.
I do question the title as I am not sure convinced that the authors are describing centriole length control per se – after all the microtubule length extension occurs at the distal end of what looks like an (almost) normal centriole.

Thank you for pointing this out. We have revised the title to "Nek1 Defines a New Branch of Centriolar Microtubule Length Control Parallel to CP110-Cep97"

In perhaps the same vein, the authors might like to note a series of papers from Benedicte Durand's lab describing how the absence of the transition zone results in extension of centriolar microtubules in the male germ line. The authors might like to discuss the relevance of such studies to their current findings.

Thank you for bringing these studies to our attention. We have now incorporated the work from Benedicte Durand's lab in the Discussion (page 17 and 18), highlighting that loss of transition zone/ciliary cap function can lead to premature and aberrant axonemal/centriolar microtubule extension in *Drosophila* male germ line, and discussing how this may relate to the centriolar microtubule hyperelongation phenotype observed in *NEK1*-deficient cells.
